# A Java-based Electronic Healthcare Record Software for Beta-thalassaemia

**DOI:** 10.2196/jmir.3.4.e33

**Published:** 2001-12-26

**Authors:** S Deftereos, C Lambrinoudakis, P Andriopoulos, D Farmakis, A Aessopos

**Affiliations:** ^1^First Department of Internal MedicineUniversity of AthensMedical SchoolLaiko HospitalAg. Thoma 17, 11527AthensGreece; ^2^Department of Information and Communication SystemsUniversity of the AegeanKarlovasi83200 SamosGreece; ^3^General Hospital of SpartiSpartiGreece

**Keywords:** Medical records systems, computerized, computerized medical record, beta-thalassaemia, delivery of health care, automatic data processing

## Abstract

**Background:**

Beta-thalassaemia is a hereditary disease, the prevalence of which is high in persons of Mediterranean, African, and Southeast Asian ancestry. In Greece it constitutes an important public health problem. Beta-thalassaemia necessitates continuous and complicated health care procedures such as daily chelation; biweekly transfusions; and periodic cardiology, endocrinology, and hepatology evaluations. Typically, different care items are offered in different, often-distant, health care units, which leads to increased patient mobility. This is especially true in rural areas. Medical records of patients suffering from beta-thalassaemia are inevitably complex and grow in size very fast. They are currently paper-based, scattered over all units involved in the care process. This hinders communication of information between health care professionals and makes processing of the medical records difficult, thus impeding medical research.

**Objective:**

Our objective is to provide an electronic means for recording, communicating, and processing all data produced in the context of the care process of patients suffering from beta-thalassaemia.

**Methods:**

We have developed - and we present in this paper - Java-based Electronic Healthcare Record (EHCR) software, called JAnaemia. JAnaemia is a general-purpose EHCR application, which can be customized for use in all medical specialties. Customization for beta-thalassaemia has been performed in collaboration with 4 Greek hospitals. To be capable of coping with patient record diversity, JAnaemia has been based on the EHCR architecture proposed in the ENV 13606:1999 standard, published by the CEN/TC251 committee. Compliance with the CEN architecture also ensures that several additional requirements are fulfilled in relation to clinical comprehensiveness; to record sharing and communication; and to ethical, medico-legal, and computational issues. Special care has been taken to provide a user-friendly, form-based interface for data entry and processing.

**Results:**

The experience gained through the use of JAnaemia in 4 Greek hospitals reveals a significant contribution towards (1) improvement of the quality of the data being recorded, since data entry is guided by appropriate forms, (2) easier cooperation between physicians, who share a common information repository, and (3) increased processing capabilities, which facilitate medical research.

**Conclusions:**

JAnaemia appears to be a useful tool, which can improve the quality of care offered to beta-thalassaemic patients in Greece.

## Introduction

Beta-thalassaemia is a hereditary disease, which results from a mutation in the genes that are responsible for the production of hemoglobin. The hemoglobin found in healthy persons is substituted by a nonfunctional protein, thus leading to severe anemia, the onset of which usually lies between the fourth and sixth month of life. Untreated severe beta-thalassaemia is uniformly fatal in childhood. Life can only be prolonged by periodic blood transfusions [1]. Unfortunately, transfusions overload patients with iron, which deposits on virtually all organs causing significant damage. Heart failure due to iron deposition on the heart, diabetes mellitus due to its deposition on the pancreas, and hepatic failure due to its deposition on the liver are only a few of the possible complications. As a result, patients suffering from beta-thalassaemia need to receive continuous chelation treatment, in order to remove the excess of iron from their body, as well as periodic hematology, cardiology, endocrinology, and hepatology evaluations. The content of these evaluations ranges from simple laboratory tests, such as complete blood count or oral glucose tolerance test, to complicated laboratory and imaging studies, such as heart and liver MRI (Magnetic Resonance Imaging) scans.

Evidently, the care process of patients suffering from beta-thalassaemia involves continuous and complex care procedures, which produce a large volume of diverse data. This makes the management of the current paper-based patient records cumbersome. The problem is further complicated by the fact that all required services are rarely offered by a single health care unit. Typically, different services are offered in different, often-distant, units, each of which maintains a separate record for each patient. Because there are currently no means of linking the individual records together, the most-common way of communication between health care professionals is through patients themselves. This often results in loss of data and in communication of inaccurate or erroneous information.

The prevalence of beta-thalassaemia is high in countries around the Mediterranean basin. It is also found in central Africa and Southeast Asia [2]. In Greece, it constitutes an important public health problem. We have therefore undertaken the development of an Electronic Healthcare Record (EHCR) software, called JAnaemia, to support the systematic collection of information related to beta-thalassaemic patients, to allow communication of patient records between the units participating in their care process, and to facilitate medical research. The software was developed in cooperation with 4 Greek hospitals and has been used in their everyday practice since late 2000. The software is based on Java [15,16] technology (see the Implementation section of Methods and in Discussion).

The complexity and diversity of the information recorded in patient records is one of the major issues with which contemporary research is concerned [3,4,5,6,7,8]. This is especially true in the domain of beta-thalassaemia, due to the inherent difficulties discussed above. JAnaemia builds upon the EHCR architecture proposed by the European Standardization Committee (CEN) in the ENV 13606:1999 standard [9,10,11,12]; in order to cope with the diversity of EHCR contents, the CEN architecture defines the building blocks from which patient records are constructed and the rules by which these can be put together, but imposes no restrictions upon the actual contents of individual records.

ENV 13606:1999 has already been implemented in a commercial product [29], which the authors have extensively used in the past, while a number of partial implementations have been produced by research groups [14]. The need for a new CEN-based EHCR application was dictated by 3 main facts:

1. Although ENV 13606:1999 defines a generic mechanism for specifying how patient record contents should be displayed, it does not further elaborate on presentation issues, allowing developers to customize them at will. Our previous experience, on the other hand, suggests that form-based data-entry Graphical User Interfaces make EHCR applications more user friendly and thus more attractive to physicians. It was therefore deemed necessary to produce a specialized version of the generic ENV 13606:1999 mechanism, in order to allow the contents of CEN-based patient records to be presented in a form-like manner.
2. Patient records, according to CEN, are composed of *Data Items*, the names of which may be specified in the form of codes retrieved from medical terminologies (see the Implementation section of Methods for a more-detailed description of *Data Items*). Existing CEN-based applications utilize the terminology produced by the Good European Health Record Project [4,7]. The authors have many times found this terminology limited, regarding the wealth of medical terms it contains and its capability of expressing the concepts pertaining to several clinical domains. This limitation is particularly important in the domain of beta-thalassaemia, due to the complexity of the health care items it involves and the number of medical terms that are necessary for recording and processing all relevant information. Such terminology limitations become critically dominant in the Greek language, where small variations in the syntax or grammar of a word may result in large variations in its meaning. The above-mentioned issues implied the need for a beta-thalassaemia-specific terminology and for an EHCR software capable of managing it. Our intention is to compare this custom terminology to currently-existing ones, in an attempt to draw conclusions on its suitability for beta-thalassaemia (see Discussion).
3. One of the user requirements for any EHCR software that would be utilized for maintaining the records of patients suffering from beta-thalassaemia was to support advanced data-processing capabilities, thus assisting health care professionals in their everyday work. Such functionality is not described in ENV 13606:1999; it was thus decided to define a generic data-processing mechanism for CEN-based EHCR applications (see the Implementation section of Methods).

Our initial goal was to develop a clinical system capable of supporting health care professionals in all data management tasks pertaining to beta-thalassaemia. However, given the dependence of our work on ENV 13606:1999, we have been able to draw conclusions on the suitability of the standard for beta-thalassaemia and to propose possible enhancements (see Discussion).

In the Design Objectives section of Methods, we present the design objectives of JAnaemia. In the Implementation section of Methods, we describe the basics of the CEN architecture and we present in detail the JAnaemia software. In Results, we present the current status of the Project. In Discussion, we present our conclusions and provide pointers to further work.

## Methods

### Design Objectives

Laiko is one of the major Greek hospitals; it is located in the capital, Athens. Its First Department of Internal Medicine has been responsible since 1980 for the cardiology follow-up of approximately one thousand patients suffering from hemoglobinopathies, including beta-thalassaemia. Although most patients live in Athens, several originate from other parts of Greece. The Thalassaemia Unit of the Ag. Sophia Children's Hospital, which is also located in Athens, is responsible for providing transfusion and chelation services to the main bulk of beta-thalassaemic patients that live in the capital. These patients constitute the majority of the Greek patients suffering from the disease. Similar services are offered by the Thalassaemia Unit of the Hospital of Korinthos to patients living in the city of Korinthos, which is approximately 100 kilometers away from Athens, and in the rural areas nearby. The pediatric clinic of the Hospital of Sparti is responsible for the management of patients living in Southeastern Pelloponese, a region approximately 300 kilometers away from Athens.

Specialized services, such as MRI studies and stress tests, are offered by other independent health care units in Athens. While these are easily accessible by the citizens of Athens, this is not the case for patients living in Sparti or Korinthos.

To provide an efficient way to cope with the continuously-growing issues of information storage, retrieval, and processing, starting in early 1998 a pilot EHCR application was developed and introduced in the everyday practice of the First Department of Internal Medicine of the Laiko hospital. In the early releases of the software, medical information was entered via static forms, which contained predetermined cardiology data. It was not possible to modify the form contents according to the needs of individual patients or health care professionals. Although this pilot project has successfully met its initial goals, it soon became apparent that considerable problems would arise if we decided to enrich patient records with information originating from specialists other than cardiologists. More specifically, it would be extremely difficult to modify the application in a way suitable for accommodating any of the other data that are frequently produced during the care process of patients suffering from hemoglobinopathies. Communication of EHCRs between different specialists would be an equally-difficult problem.

It was therefore decided to redesign the application, taking into account the following issues:

1. The expected diversity of record contents; patient records should be allowed to contain information pertaining to any of the medical specialties and health care procedures relevant to beta-thalassaemia.
2. The diverse needs of health care units or health care professionals; different units offer varying ranges of services, while health care practices may also vary. The software should be customizable to the needs of individual users.
3. Ease of use and user friendliness; the form-based user interface, implemented in the pilot EHCR application, has been positively evaluated by physicians and it should therefore be utilized in the new application.
4. Automated data processing; the care process of patients suffering from beta-thalassaemia involves clinical decisions that are based on the results of laboratory tests, or on parameters derived as complicated functions of these results. All calculations required for the parameters are currently made by hand; they should be automated by the software.
5. Record sharing, within the context of a single unit or between units; contributions made to the record of each patient by various health care professionals should be communicable to all other professionals involved in the patient's care process. Since patient mobility has increased within the European Union, ethical, medicolegal, and automatic-translation issues should be taken into account. In this context, off-line methods for exchanging medical data between professionals should also be considered. An example is the support of personalized patient smart cards for storing a "minimal medical set" for the card owner.
6. Scalability; the software should be capable of running on different machines and operating systems, to allow different hardware and network configurations, according to the size of the units in which it operates.
7. Performance; the technology that will be employed for the development of the application must be appropriate for a hospital environment, where a significant number of users should be allowed to concurrently access the patient medical records and other supported functionality, without causing a deterioration of the application's performance or unavailability of the information.
8. Automatic integration of laboratory results into medical records; although this is a design objective for the new application, it has not been implemented in the current version since the participating hospitals do not have the necessary technical infrastructure for exporting laboratory data in electronic form.

### Implementation

#### Electronic Healthcare Record Architecture

As mentioned in the Design Objectives section of Methods, the capability to manage diverse patient-record contents and the flexibility to customize the application to the needs of individual users are 2 major design objectives. Such features are provided by the Electronic Healthcare Record architecture proposed by CEN in the current European standard, ENV 13606:1999. In addition, the CEN architecture takes into account computational, educational, and medicolegal issues related to patient records, as well as issues of sharing, of clinical analysis, and of clinical comprehensiveness [4,13]. JAnaemia is, therefore, based on this standard, an overview of which we provide in the current subsection.

ENV 13606:1999 defines that Electronic Healthcare Records consist of *Compositions* created during the various instances of health care service provision. Each *Composition* contains all data recorded at one place and time of care delivery, in a single session with a particular health care professional [9]. During a patient's lifetime, numerous *Compositions* may be created at different health care institutes, for different reasons. All these are uniquely identified and can be linked together to constitute the patient's lifelong EHCR. *Compositions* are the minimum groupings of patient-record data that can be safely transferred between different locations, without altering the meaning initially conveyed or violating any medicolegal rules that may apply to EHCR communication [9,17].

*Compositions* comprise *Data Items (DIs)*, either single or grouped together in *Clusters*. *DIs* are the smallest structural units into which the content of EHCRs can be broken without losing its meaning. *DI* clusters provide an aggregation mechanism for the representation of compound concepts. They consist of single *DIs*, linked together in a tree-like structure [9,17].

**Figure 1:**
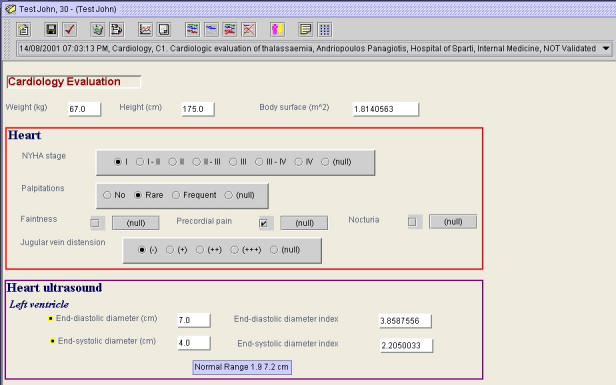
An extract from a Composition of a patient record

Figure 1 illustrates the above concepts. It presents an extract from the record of *Test John*, who is 30 years old. The details of the particular *Composition* are given in the combo box, directly under the toolbar; it was created on 14/08/2001, at 07:03:13 pm, by Dr Andriopoulos Panagiotis, at the department of Internal Medicine of the Hospital of Sparti and it contains a Cardiology evaluation. The combo box in Figure 1 contains a combination of different types of information, yet all this information is combined in a single string. The user can select from a drop-down list of such strings.

The reader can see that all the contents of the composition presented in Figure 1 lie under the heading "Cardiology Evaluation". *Headed Sections*, as defined in ENV 13606:1999, are subdivisions of compositions which are used to group entries with a common theme or which are derived through a common health care process [9]. *Headed Sections* often convey the situation in which the information was gathered (such as "cardiology evaluation") or the time relationship, place relationship, or person relationship the information has to the patient (such as patient history, management plan, or family history), without placing any constraints on the data they in turn contain [4,13,17].

Information on the patient weight, height, and body surface is recorded in the single *DIs* *Weight*, *Height*, and *Body surface* respectively, immediately below the heading. Although they are located under the *Headed Section* Cardiology Evaluation, there are no other constrains pertaining to those three *DIs*. Heart ultrasound results, on the contrary, are grouped under the item *Heart Ultrasound*, thus forming a *Cluster*. *DIs* *End-diastolic diameter*, *End-systolic diameter*, *End-diastolic diameter index*, and *End-systolic diameter index* are now perceived as conveying more-detailed information on the patient's *Heart ultrasound* and, more specifically, on its results pertaining to the *Left ventricle*. Although it is allowed to remove the *Weight*, *Height*, or *Body surface DIs* from the Headed Section, it is not allowed to remove any of the *DIs* that constitute the *Heart ultrasound* cluster; it is only allowed to remove the entire cluster.

The *Composition* in Figure 1 also includes selected information from the history and the clinical examination of the particular patient, grouped in the *Heart* cluster. The *Heart* and *Heart ultrasound* clusters and the *Weight*, *Height*, and *Body surface* single *Data Items* were all created at the same date and time, at the same place, by the same health care practitioner. They convey information on the health status of the patient at a particular point in time. For this reason, they are grouped together, thus forming a *Composition*.

*Data Items* are further qualified by their attributes, which convey all relevant information; the name of a *Data Item* is recorded in the *Component Name* attribute; its data type is recorded in the *Data Type Item Reference* attribute, and its actual content in the *Data Item Content* attribute. *Data Item* names may be specified in the form of codes retrieved from medical terminologies, such as SNOMED [18], UMLS [19] and the ones produced by the GEHR (Good European Health Record) [4] and GALEN Projects [20,21].

The *Component* *Name* attribute of the *End-systolic diameter* item, presented in Figure 1, has the value *End-systolic diameter*(as one might expect), while its *Data Item Content* attribute has the value *4*. *DIs* can be further qualified by the *units* in which their contents are measured and their *range of normal values*. In our example, the units are *centimeters* *(cm)* and the normal range *1.9* to *7.2 cm*, as indicated by the tooltip in Figure 1. See Figure 1 for more examples and to the ENV 13606:1999 CEN standard for a complete account of the *Data Item* attributes.

JAnaemia implements all the above-mentioned concepts. CEN defines, in addition, a number of architectural sub-components, which are partially implemented in our work. They, too, are presented in detail in ENV 13606:1999.

#### Record Sharing and Communication

Efficient record sharing is of utmost importance in the domain of beta-thalassaemia, mainly because health care services are offered by multidisciplinary teams, usually scattered over several departments of even different units. It is, therefore, required that compositions created at the various places of care provision are communicated to all involved health care professionals.

The CEN architecture describes rules for distributing Electronic Healthcare Records, called *distribution rules*. *Distribution rules* are logical concepts or rules intended to convey intent for and govern the distribution of the EHCR components (the *Data Items*, *Clusters* and *Compositions* discussed in the Electronic Healthcare Record Architecture subsection of the Implementation section of Methods are examples of such components). *Distribution rules* constitute a controlling mechanism, enabling access to and/or further distribution of the components to which they are attributed. Distribution rules define, among other things, *who* should have access to a record component, *when* this access should be granted, *where*(ie, to which countries and/or health care units) and for *what purposes* the component may be distributed. CEN defines the general principles that govern EHCR distribution. Specific implementations are left to the developers of EHCR software [11].

CEN also describes a set of messages that implement the above-mentioned distribution rules and enable the electronic transfer of health care record information [12]. They have the form of XML documents and they are designed to allow the exchange of EHCR information between different types of clinical information systems.

The EHCR architecture presented in the Electronic Healthcare Record Architecture subsection of the Implementation section of Methods and elaborated on in ENV 13606:1999, together with its accompanying distribution rules and exchange messages, ensure that all pertinent ethical and legal issues are taken into consideration when EHCRs are transferred between health care professionals, working either in different units or in the same unit.

On the programming level, JAnaemia is based on the Java [15,16] technology. As mentioned in Introduction and the Design Objectives section of Methods, the software operates at departments of 4 different hospitals, of varying size and requirements. As the Project expands, smaller units (such as primary care centers) and bigger units (such as entire hospitals) may be involved. JAnaemia should be capable of running on a variety of operating systems, supporting different hardware and network configurations, according to the size of the involved units. While a single Intel-based PC [27] running Microsoft Windows 2000 [30] may be sufficient for a primary care center, a network of servers based on more-powerful processors, such as Sun's UltraSPARC [28], might be required for larger units. The portability features of Java make it an ideal choice for this purpose.

An additional reason for relying on Java is the rapidly-increasing use of Internet appliances. Several research groups have found the use of palmtop computers beneficial for data entry [31,32]. Java and the EmbeddedJava application environment [33] constitute one of the major operating environments for such devices. Since future work will focus on producing a version of JAnaemia for internet appliances, it was considered appropriate to develop it in Java from the very beginning.

EHCRs are stored in a central repository, currently implemented as a relational database maintained by Microsoft's SQL Server v7.0 [22]. SQL Server v7.0 was initially selected on the basis of its high performance [22]; yet the selection of the database engine may vary among health care units, depending on the their size, on the expected workload and, on the software and hardware configuration on which JAnaemia operates in each case.

Physician workstations run JAnaemia as a client that connects to the central repository via JDBC (informally, Java Database Connectivity) [23] - a method of interfacing Java software with SQL databases - and thus allows users to access the patient records. This architecture supports record sharing at the level of a single department or health care unit. In this context, individual workstations are linked to the database server via local intranets [24]; new compositions are stored in the EHCR repository, as parts of the patient records to which they pertain. In this manner, all members of the unit or department have direct access to the entire EHCR repository. It should be stressed that compositions are stored in the repository together with their associated distribution rules. This ensures that appropriate access and transfer permissions are enforced, even in the context of a single health care unit or department. Thus, from the point of view of efficient and secure record sharing, accessing an EHCR from a common repository or over longer distances, via exchange messages, are equivalent.

Although the record sharing architecture (described in the paragraph directly above) method of sharing could be used over longer distances, via Internet for example, experience shows that it cannot reach the desired degree of efficiency. JAnaemia makes heavy use of JDBC and network resources and requires fast and reliable network connections. For this reason, a separate EHCR exchange mechanism is being developed, to allow communication of EHCRs between distant health care units. Complete patient records or record extracts are exported in the form of messages, implemented as XML documents, according to the Document Type Definitions (DTDs) provided by CEN [12]. They can then be transferred to remote sites over any available communication medium. Upon reception, they are imported to the local EHCR repository and from then on, they can be processed via JAnaemia. Work is under way to develop smart-card and Internet-based record-exchange solutions. In view of these future developments, the compatibility of Java with the JavaCard technology [34] was an additional reason for selecting Java as the basis for our work.

Currently, JAnaemia operates as a stand-alone application, since the participating hospitals do not feature an integrated Information System. However, compatibility with hospital information systems that may be installed in the future will be achieved via the above-mentioned message-based EHCR exchange mechanism, the design of which allows the communication of information between different types of systems [12].

#### Miscellaneous Functionality

As suggested by the authors' experience, form-based data-entry Graphical User Interfaces make EHCR applications more user-friendly and thus more attractive to physicians. Yet, ENV 13606:1999, as mentioned in Introduction, is not specific on how presentation issues should be handled. Previous implementations of the standard do not provide form-based data-entry interfaces. It was therefore deemed necessary to implement such an interface in JAnaemia. For this purpose, it should be possible to use the standard controls supported by the various operating systems, such as check boxes, text boxes, combo boxes, and groups of radio buttons, for entering data in *Data Items*(see the Electronic Healthcare Record Architecture subsection of the Implementation section of Methods for a description of *Data Items*). To provide this functionality, we have defined 3 *Data-Item* attributes in addition to the ones already defined by CEN:

(1) The *Display Type Item Reference* attribute conveys information on the way *Data Items* should be displayed. It is complementary to *Data Type Item Reference*.

JAnaemia supports simple and compound data types. Simple data types are used to store plain numeric, text, date, and time values. Compound data types are used to store coded terms, originating from medical terminologies, as well as combinations of the latter with any of the simple data types. A separate data type is used to store multimedia objects. Basic type checking is performed on the contents of *Data Item* s to ensure their compatibility with their associated data types. This includes the validation of date-time values.

Each data type may be associated with certain display types: numeric *Data Item* s may be presented as text boxes, check boxes, toggle buttons, groups of radio buttons, or combo boxes; date-time *Data Items* may be presented as text boxes or combo boxes, while text and compound data types may be presented as combo boxes or specialized data entry windows.

**Figure 2:**
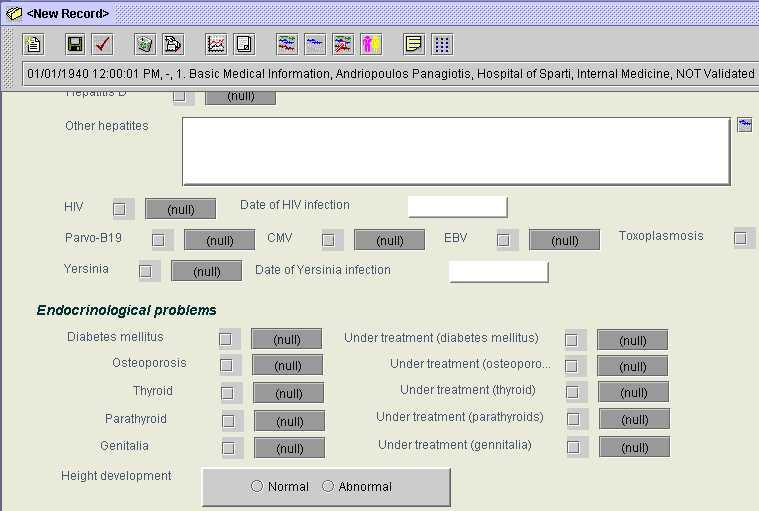
An extract from a Basic Medical Information Composition

Figure 2 presents examples of *Data Items* displayed in different ways. The *Height development* *Data Item* is displayed as a group of radio buttons, from which the user can make a selection. *Date of HIV infection* and *Date of Yersinia infection* items are expected to receive a date value and are displayed as plain text boxes. *Other hepatites* may receive a string value and is therefore presented as a large edit window. *Diabetes mellitus*, *Osteoporosis* and *Thyroid* may be true or false and are presented as checkboxes.

(2). the *X Position* and (3) the *Y Position* attributes define the location on the data-entry window where a given item should appear. The data-entry window is the one in which *Composition* contents are displayed. It is an inner, scrollable window, thus allowing the system to automatically adjust to different screen sizes; *Composition* contents that lie outside the visible screen area can easily be scrolled in the window.

This absolute positioning of *Data Items* allows users to combine them in complex arrangements, thus creating custom data-entry forms according to the users' own needs and tastes. It is more flexible than relative positioning, as it provides additional freedom in defining item locations.

It was a user requirement that JAnaemia provides a mechanism for automatically assigning values to *Data Items*, by processing related items of the patient record, referring either to the same or to different *Compositions*. This would both facilitate data entry and serve as an elementary decision-support tool. It should be possible, for example, to calculate the patient *Body Surface* as a function of the patient's *Weight* and *Height*. Such functionality has been supported through a fourth *Data Item* attribute, called *Macro-directive*. The *Macro-directive* attribute contains macro-directives which specify the automatic-processing behavior *Data Items* should exhibit ( *Macro-directives* are defined, in the context of our work, as statements capable of retrieving and processing the contents of *Data Items*, and of assigning values to them). See Discussion for further explanations and more examples.

## Results

### Status Report

JAnaemia is generic-purpose EHCR software that can be used in all medical domains. New *Compositions* added to patient records are completely empty. Their contents need to be defined by users and can vary according to individual needs. *Compositions* referring to the Cardiology evaluation of patients, for example, should contain all *Data Items* required for recording the information produced by the patient's history and physical examination, by the patient's ECG and cardiac ultrasound examination, etc. *Compositions* referring to the patient's Endocrinology evaluation, on the other hand, should contain *Data Items* concerning the patient's thyroid and sex hormones, bone density measurements, etc. To facilitate data entry, JAnaemia allows the definition of sets of *Data Items*(henceforth called *data item sets*) that refer to a common item of care and can be utilized as a single entity. The contents of the *Composition* in Figure 1 are part of the Cardiology Evaluation *data item set*, while those of the *Composition* in Figure 2 are part of the Basic Medical Information *data item set*. *Data item sets* are equivalent to data entry forms, which makes their role in facilitating data entry crucial.

Ten *data item sets* have been created, in cooperation with the above-mentioned hospitals, to cover the information-recording and information-processing needs of beta-thalassaemia, namely: (1) a set for entering administrative information (patient name, sex, age, date and place of birth, etc.), (2) a Basic Medical Information set, (3) a Chelationset, (4) a Transfusion set, (5) an Endocrinology evaluation set, (6) a Cardiology evaluation set, (7) a Hepatology evaluation set, (8) an Annual transfusion summary set, (9) an Annual chelation summary set, and (10) an Annual report on the L1 oral chelation treatment.

These sets have been used in the everyday practice of the participating hospitals since late 2000. Although the sets are roughly the same in all installations, small differences among them reflect the slightly different health care practices followed in each hospital. For example, the contents of the Hepatology Evaluation set in the Ag. Sophia Children's Hospital is different from the contents of the Hepatology Evaluation set in the hospitals of Korinthos and Sparti. The former is a tertiary hospital, with access to a broad range of laboratory tests that physicians routinely perform and store in patient records. Some of these tests are not readily available at Korinthos and Sparti; physicians at these hospitals therefore use a simpler Hepatology Evaluation *data item set* than the one used at Ag. Sophia Children's Hospital. The same goes for the other sets. In addition, Ag. Sophia Children's Hospital participates in a clinical trial on the new L1 oral chelation treatment. In the context of this trial, physicians routinely check for side effects of the L1 pill and perform additional tests that are part of a predetermined research protocol. Two additional *data item sets* have been created to cope with the increased recording and processing needs: (1) a Side effects of the L1 pill set and (2) an Additional Laboratory Investigations set.

The number of *data item sets* and their contents can vary among health care units, departments, or even health care professionals. Users may customize them according to their own needs. Customization is performed through the GUI (Graphical User Interface) of JAnaemia and does not involve modifying the software or the structure of the underlying database. As soon as they are created, custom *data item sets* may be used to define the contents of the new *Compositions* that are added to patient records. No matter how diverse these contents are, JAnaemia can process them and store them in the EHCR repository, in the uniform way defined in ENV 13606:1999. *Compositions* are stored together with their *distribution rules*(described in the Record Sharing and Communication subsection of the Implementation section of Methods) and can be directly shared with users accessing the same repository or with remote users, via exchange messages.

The current use of JAnaemia involves recording and processing of all care items related to the beta-thalassaemic patients treated at the 4 participating hospitals. For example, the software is used by the 6 physicians serving at the Thalassaemia Unit of the Ag. Sophia Children's Hospital. The unit is responsible for the follow up of about 500 patients, 30 of whom are part of the L1 clinical trial. Each patient receives transfusions approximately 2 times every month and undergoes laboratory examinations at regular intervals. During a normal day, the unit treats approximately 80 patients.

At the Laiko hospital, JAnaemia is used by 6 physicians of the First Department of Internal Medicine. The department is responsible for the cardiology evaluation of approximately 1000 patients suffering from various hemoglobinopathies, including beta-thalassaemia. Patients are typically examined once or twice a year.

The Thalassaemia Unit of the hospital of Korinthos provides transfusion, chelation, and other follow-up services to 50 patients, which on average visit the department twice a month. JAnaemia is used by 3 of the physicians serving there, as well as by 4 nurses. Efforts are under way to engage the nursing stuff of the other hospitals in the use of the software.

The hospital of Sparti is responsible for 10 patients, which are treated by 2 physicians of the Pediatric department.

## Discussion

### Electronic Healthcare Record architecture and clinical functionality

JAnaemia was found to facilitate clinical work by allowing patients' records to be well organized and easily accessible as well as by offering advanced processing functionality. The latter includes the macro-directive mechanism, presented in the Miscellaneous Functionality subsection of the Implementation section of Methods, which assists users in entering data and provides elementary decision support, as well as statistical functions.

The macro-directive mechanism was found to be particularly useful in clinical practice, as it facilitates data entry and it provides elementary decision-support functionality. The Cardiology *data item set*, for example, includes approximately 150 automated calculations; physicians enter a number of basic parameters, produced by the cardiac ultrasound equipment, and are automatically provided with many other parameters, which are functions of the basic parameters. The cardiac mass and the left ventricular volume are calculated from the end-systolic and the end-diastolic diameters of the left ventricle, the total cardiac diameter is calculated by adding the diameters of the individual cavities, etc. The calculated parameters are valuable to cardiologists, who use them as a basis for their clinical decisions. The interpretation of an Oral Glucose Tolerance Test (OGTT) is another example. OGTT is used to diagnose diabetes mellitus. After orally administering to the patient a predefined quantity of glucose, the patient's blood glucose levels are measured every 30 minutes, for 3 hours. If blood glucose before the test starts is >= 140 mg/dl, then diabetes mellitus is diagnosed. If it is < 140 mg/dl and blood glucose measured 2 hours after the initiation of the test is >= 200 mg/dl, then again diabetes mellitus is diagnosed. If blood glucose before the test starts is < 140 mg/dl, and if at 2 hours it is >= 140 mg/dl but < 200 mg/dl, then impaired glucose tolerance is diagnosed [2]. JAnaemia allows recording the blood glucose levels during an OGTT. After data is entered, an appropriate macro is executed, to check the above-mentioned conditions. It then checks or unchecks the *OGTT indicative of glucose intolerance* and *OGTT indicative of diabetes mellitus* *Data Items* as appropriate, thus indicating to the user the results of the test.

The macro-directive mechanism is also useful for maintaining a Basic Medical Information *Composition*, which is automatically updated as new data are added to the patient record. Basic Medical Information contains a minimal set of data summarizing the patient's health status. Physicians consult it whenever they need to quickly get an overall view of the patient's problems.

The advanced data-processing functionality provided by the macro-directive mechanism was the main reason for developing JAnaemia as a thick client written in Java, an object-oriented programming language, rather than as a thin client or as a Web-based application. A thick client is software that does most of the processing required for a task, leaving little or no processing to be done on the server. A thin client is simple software that performs very little processing, leaving most of it to be done on the server. Java provides greater flexibility and ease of implementation than a Web-based application in relation to the handling of the complicated data structures and of the logic involved in such a mechanism. Furthermore, Java offers developers the opportunity to perform a substantial part of the necessary tasks in the memory of the client, thus improving the overall performance of the application, as opposed to continuously accessing the database server. In large health care units, where several users may concurrently request such processing, the performance gain is even bigger. In a thin client or Web-based alternative, most of the operations would need to be performed directly on the database server and/or in the server memory, thus quickly leading to the exhaustion of server resources and deterioration of the performance.

The CEN architecture, as suggested by the experience accumulated so far, is capable of modeling all the basic information structures pertinent to beta-thalassaemia. It also allows customization to the particularities of individual health care units (see Results).

Our proposed enhancements to the CEN architecture, described in the Miscellaneous Functionality subsection of the Implementation section of Methods, were found to be useful for clinical practice. CEN defines a *Presentation Information Data Item* attribute, which, among other things, dictates the way in which items should be displayed [9]. However, ENV 13606:1999 is not specific about how the presentation issues are to be specified. We found that our method of specifying presentation information is useful for clinical practice, as it provides a user-friendly, form-based interface that greatly facilitates data entry. Our aim is to get the method evaluated by the responsible CEN workgroup, for possible inclusion in future versions of the standard. The macro-directive mechanism for processing patient record contents, previously discussed, was also found to be a useful feature. As it is much easier to implement such mechanisms than other, more-sophisticated, data-processing and decision-support techniques, we will also investigate the possibility of having such mechanisms incorporated in future versions of ENV 13606:1999.

Other proposals on the EHCR architecture do exist. The Synapses Project [5] and the HL7 group [6] are some of the research teams working in this area. Their architectures are flexible and comprehensive as well; the CEN architecture, however, is the current European standard. The authors have been convinced of its value and believe that it should be endorsed, in order to gain wide acceptance. On the other hand, joint efforts are made by the various research groups to bridge the differences of their proposals and to converge to a common standard [35,36]. It is the authors' intent to follow these future developments of ENV 13606:1999 and to adjust JAnaemia as appropriate.

As mentioned in the Electronic Healthcare Record Architecture subsection of the Implementation section of Methods, the names of the *Data Items* comprising an EHCR may be specified in the form of codes retrieved from medical terminologies. Health care professionals, on the other hand, pose very strict requirements on the wording of patient record contents. The problem is more intense in the Greek language, in which small variations in the syntax or grammar of a word may result in large variations in its meaning. The authors have many times had difficulties in modeling various clinical domains, using the GEHR terminology, on which existing CEN-based EHCR applications rely. We have therefore decided to initially create a proprietary medical terminology for beta-thalassaemia, which would be shared by the participating units and would be capable of expressing all concepts related to the disease in the exact way demanded by the participating health care professionals. Work is under way to compare the resulting terminology to other existing ones, such as GALEN, SNOMED, GEHR, and UMLS. If any of these terminologies proves sufficient for beta-thalassaemia, it will be adopted, replacing our proprietary one. In the opposite case, the authors shall either propose amendments to existing terminologies, or investigate alternative exchange methods that are capable of communicating records based on proprietary terminologies.

An important feature that was found to facilitate medical research is the capability to export database search results in a format directly readable from widely-used statistical packages, such as SPSS [37] or SAS [38]. JAnaemia supports database searches defined by combining any number of simple criteria, as many other EHCR applications do. Users can, for example, search the EHCR repository for patients that have *total cholesterol* over 250 mg/dl and *blood pressure* over 160/100 mmHg. However, the parameters sought each time, such as *total cholesterol*, may occur more than once in each record. Statistical tests that are used to compare population parameters, on the other hand, require that only one of their values is associated with each patient. Researchers should decide whether they want to extract, for example, the last or the biggest cholesterol value. JAnaemia provides users with appropriate data-extraction options, thus automating the described task, which would otherwise have to be performed by hand.

### Technical issues

From a technological point of view, Java was found to be powerful enough for handling all storage/retrieval, processing, and user-interface related issues in an acceptable way. After several stages of optimization, today JAnaemia provides a responsive user interface and performs all basic operations, including loading, saving, and analyzing EHCRs, in a few seconds. More specifically, the following performance figures were obtained for the main application tasks, for all the user configurations described in Results:

- Data entry is performed in real time. This includes the time required to execute the macro directives relevant to the *Data Items* being edited. In some cases dozens of calculations may be performed.
- New compositions are saved in less than a second. A large composition, in our customization for beta-thalassaemia, typically contains approximately 250 *Data Items*.
- The time required to load patient records is proportional to the number of *Data Items* they contain. One of the largest EHCRs in our databases contains information on 120 transfusions, on 10 cardiology evaluations, and on 20 evaluations of other types. These were accumulated during the 10-year follow up of the particular patient. The EHCR contains a total of 10000 *Data Items* and is loaded in 25 seconds. It is important to note that a separate control is created for each loaded *Data Item*. Most EHCRs, however, are loaded in less than 10 seconds
- The time consumed during database searches exhibits large variations depending on factors such as the size of the database and the number of matching records. In the database of the Ag. Sophia Children's Hospital, which contains 500 records, a typical search takes approximately 60 seconds. This is an acceptable delay, taking into account the diversity of patient record contents, which necessitates specially-designed searching strategies, as opposed to the plain SQL [25] queries that can be utilized in EHCR applications that rely on static data entry forms

The above measurements were obtained on computers based on Intel Pentium III [27] processors, operating at 733 MHz (megahertz). According to our observations, the time consumed by the above-mentioned tasks is inversely proportional to the CPU (central processing unit) clock speed. We therefore expect that on the 2 GHz (gigahertz) Pentium IV processors that are now available, the reported times will decrease by a factor of 2.7.

Despite the large number of windows and controls it creates, JAnaemia never occupied more than 80 MB (megabyte) of memory even after long (6 hour) continuous operation. As a result, it runs smoothly on the 256 MB RAM computers that are now widely available.

Based on our monitoring, one of the most time-consuming processes in EHCR software is communication with the database server, via JDBC. The delay increases as the patient database grows larger. In CEN-based EHCR software, the problem becomes even worse; as described in the Electronic Healthcare Record Architecture subsection of the Implementation section of Methods, patient records consist of *Compositions*, which contain *Data Items*, which are in turn qualified by attributes. In early versions of JAnaemia, information on patients, *Compositions*, *Data Items* and attributes were stored in separate tables. An average sized record for beta-thalassaemia contains approximately 2500 *Data Items*, each of which is qualified by 15 attributes. It therefore corresponds to 2500 x 15 = 37 500 entries in the attributes table. We very soon realized that the latter would grow beyond bounds very fast; that fact alone would make performance suffer and would exhaust the available database space. As a result, we were forced to store *Data Item* attributes in separate fields of the *items* table, thus making it possible to store all information on an item in a single table row. Although the new database schema resulted in some redundancy, since not all attributes are required for every *Data Item*, it also led to a 10-fold increase in application performance and substantially reduced the database size.

Optimizing the design of the database schema to reduce the number of SQL statements required to retrieve EHCRs, therefore, results in immense performance gains. Keeping all frequently-accessed data in memory and performing as much processing as possible there, rather than continuously accessing the database, also increases performance. JAnaemia relies heavily on strings and string arrays for temporarily storing data; A comparison of the current version of JAnaemia with a new one, in which string handling will be implemented in C++, is planned, so that we can assess the improvement in performance, due to the enhanced string-handling capabilities of C++.

The performance of JAnaemia increased by up to 300% with the aid of the JOVE optimizing native compiler [26].

Despite the extensive arguments of software engineers against the speed and efficiency of the Java programming language, we found it to be sufficient for the needs of EHCR applications. Careful programming and appropriate database design, as described above, aided by optimizing native compilers, makes its performance acceptable for clinical use. Furthermore, it provides scalability features that are important in the diverse health care environment.

Platform independence is a Java feature that makes possible scalable designs, suitable for the needs of health care units differing in size. Currently, JAnaemia operates on stand-alone Pentium-based computers. Both JAnaemia and the database server run on the same machine. As the Greek University Network (GU-Net) and other health-care-related networks expand, all hospitals in Athens will be linked together. It will then be possible to link individual units to a common EHCR repository. This will require more-powerful database servers and, in certain cases, more-powerful workstations. Java makes it possible to directly port JAnaemia to computers based on more-powerful processors, such as Sun's UltraSPARC series. It also facilitates its porting to Internet appliances, which can provide mobile solutions to health care professionals.

### Conclusions

Participating health care professionals quickly accustomed themselves to the software and now find it superior to their initial, paper-based records, in terms of both efficiency and time consumption. The form-based GUI, the macro-directive data-processing mechanism and the direct link to statistical packages, described in the Electronic Healthcare Record architecture and clinical functionality section of Discussion, were the main factors that contributed to its acceptance. These conclusions are currently based on personal communication with the involved health care professionals; formal user-satisfaction surveys are planned during the next phases of the Project.

To conclude, JAnaemia appears to be a useful tool for health care professionals involved in the care process of patients suffering from beta-thalassaemia. It is based on the European Standard Electronic Healthcare Record architecture and it has several features that render it user-friendly and provide it with advanced data-processing functionality. It possesses all the prerequisites for EHCR exchange while specific communication solutions are currently under development. We believe that JAnaemia can improve the quality of care offered to beta-thalassaemic patients in Greece.

## Abbreviations

CEN: European Committee for Standardization
DI: Data Item, as defined in the ENV 13606:1999 CEN standard
DTD: Document Type Definition
EHCR: Electronic Healthcare Record
GEHR: Good European Health Record
GU-Net: Greek University Network
GUI: Graphical User Interface
JDBC: Java Database Connectivity (informal; JavaSoft, the maker of JDBC, does not consider it to be an abbreviation)
MB: Megabyte
MRI: Magnetic Resonance Imaging
OGTT: Oral Glucose Tolerance Test
XML: Extensible Markup Language

## Multimedia Appendix

Downloadable presentation " A Java-based Electronic Healthcare Record for beta-Thalassaemia "
