# Supplementary material for: A Java-based Electronic Healthcare Record Software for Beta-thalassaemia
Source: J Med Internet Res. 2001 Dec 26;3(4):e33. doi: 10.2196/jmir.3.4.e33 (PMC1761918; doi:10.2196/jmir.3.4.e33)
Supplement: Supplementary file 1 [file jmir_v3i4e33_app1.ppt]

## Slide 1
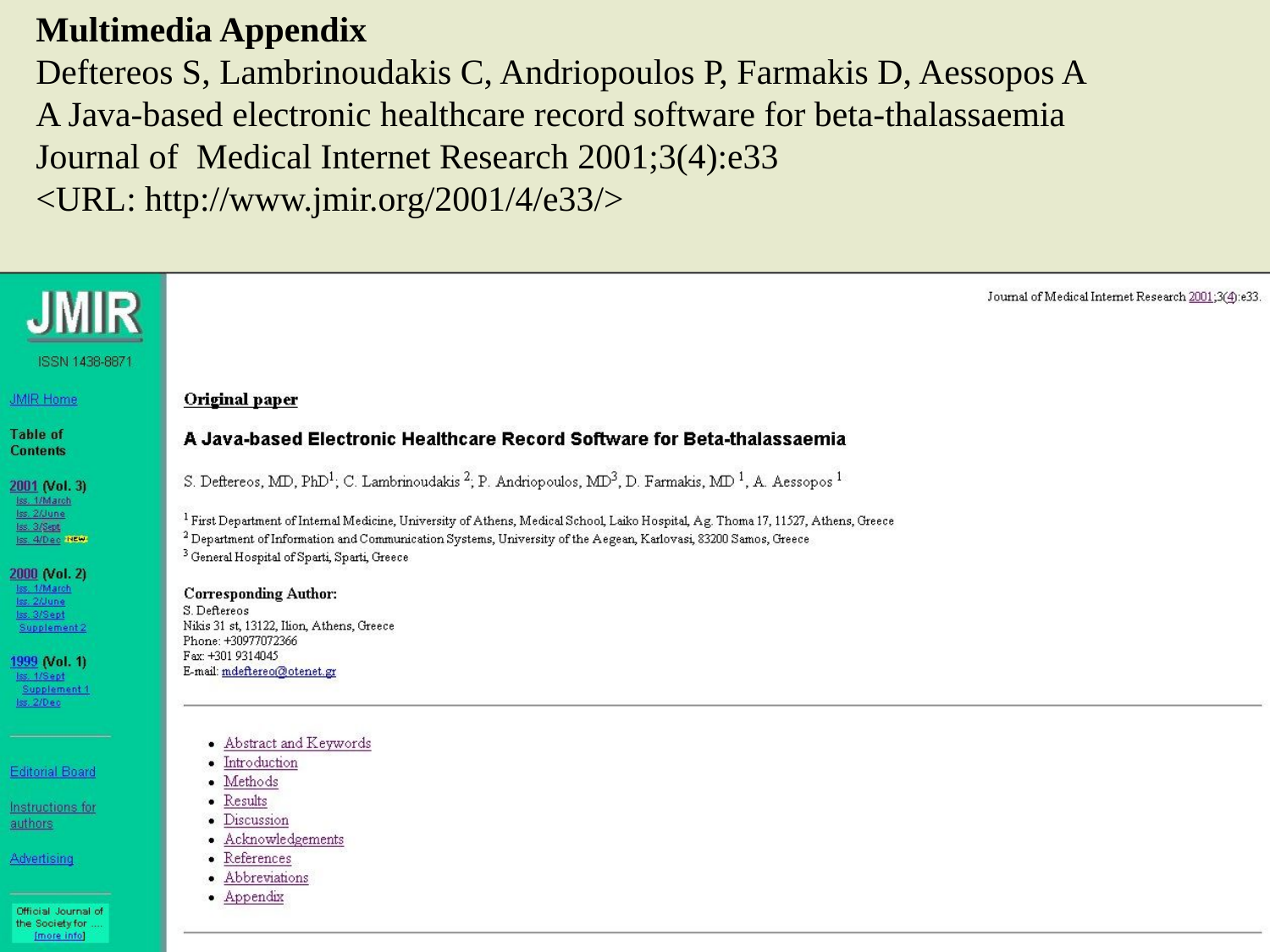

Multimedia Appendix
Deftereos S, Lambrinoudakis C, Andriopoulos P, Farmakis D, Aessopos A
A Java-based electronic healthcare record software for beta-thalassaemia
Journal of  Medical Internet Research 2001;3(4):e33
<URL: http://www.jmir.org/2001/4/e33/>

## Slide 2
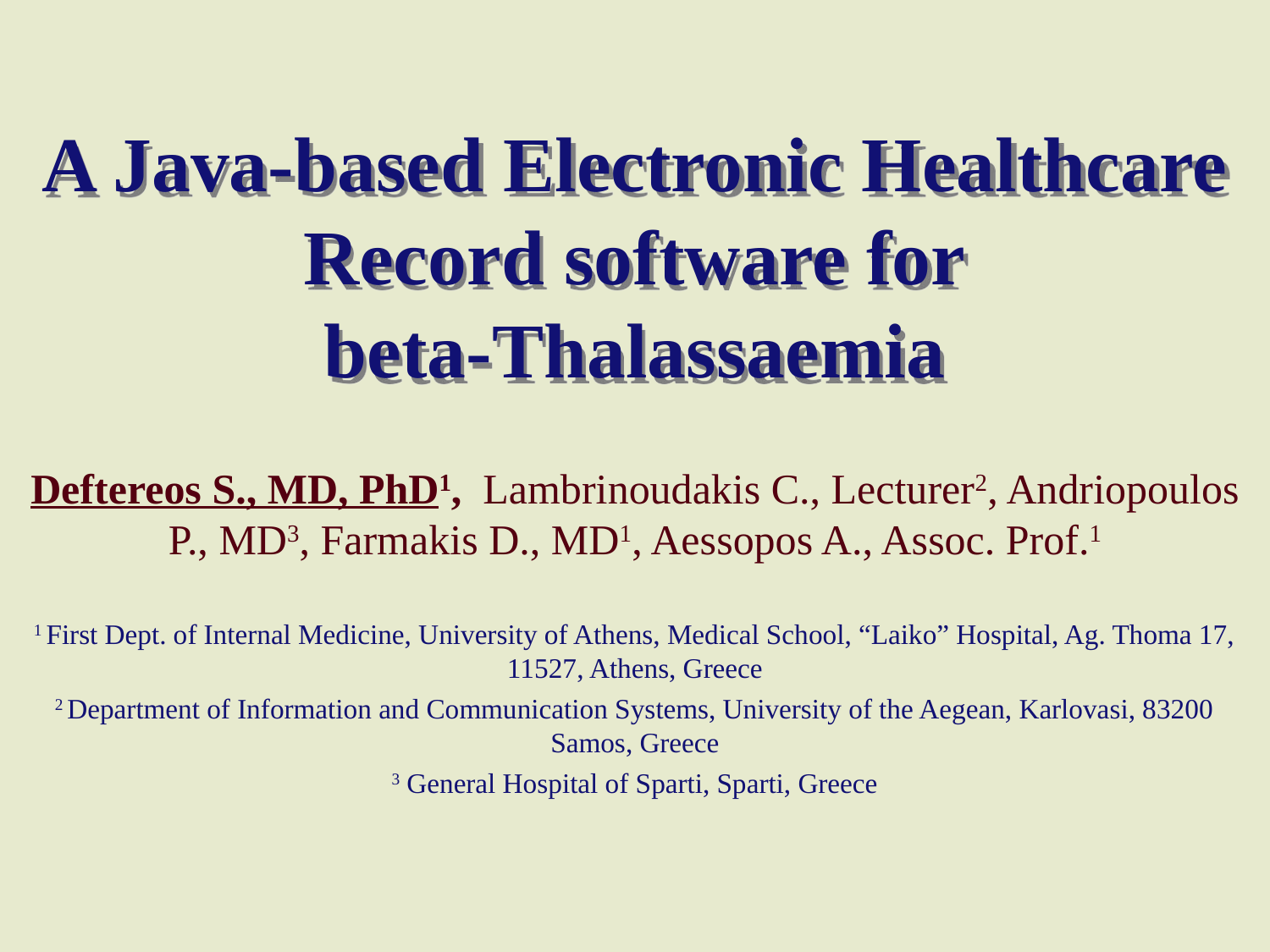

# A Java-based Electronic Healthcare Record software for beta-Thalassaemia
Deftereos S., MD, PhD1, Lambrinoudakis C., Lecturer2, Andriopoulos P., MD3, Farmakis D., MD1, Aessopos A., Assoc. Prof.1
1 First Dept. of Internal Medicine, University of Athens, Medical School, “Laiko” Hospital, Ag. Thoma 17, 11527, Athens, Greece
2 Department of Information and Communication Systems, University of the Aegean, Karlovasi, 83200 Samos, Greece
3 General Hospital of Sparti, Sparti, Greece

## Slide 3
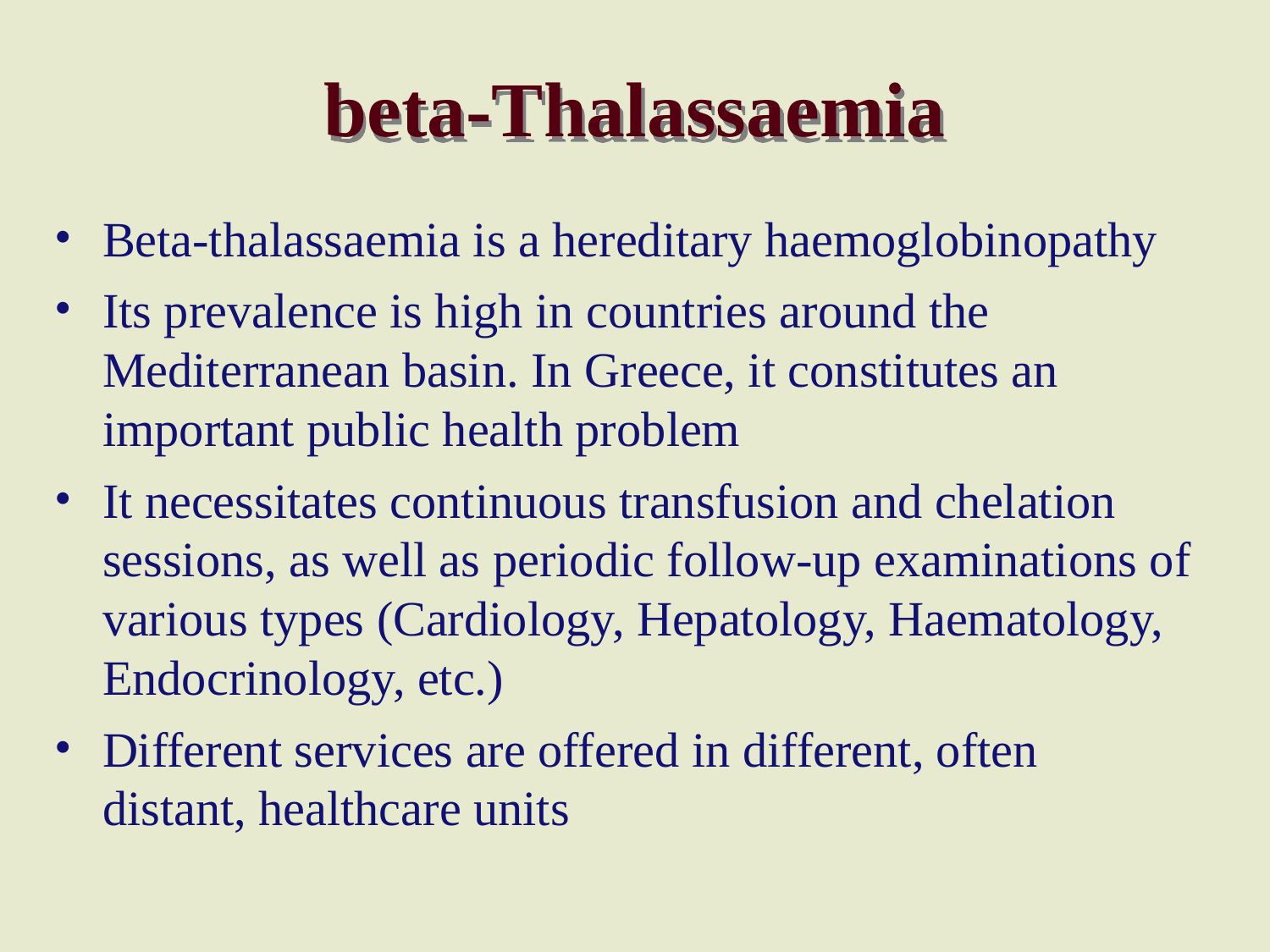

beta-Thalassaemia
# Beta-thalassaemia is a hereditary haemoglobinopathy
Its prevalence is high in countries around the Mediterranean basin. In Greece, it constitutes an important public health problem
It necessitates continuous transfusion and chelation sessions, as well as periodic follow-up examinations of various types (Cardiology, Hepatology, Haematology, Endocrinology, etc.)
Different services are offered in different, often distant, healthcare units

## Slide 4
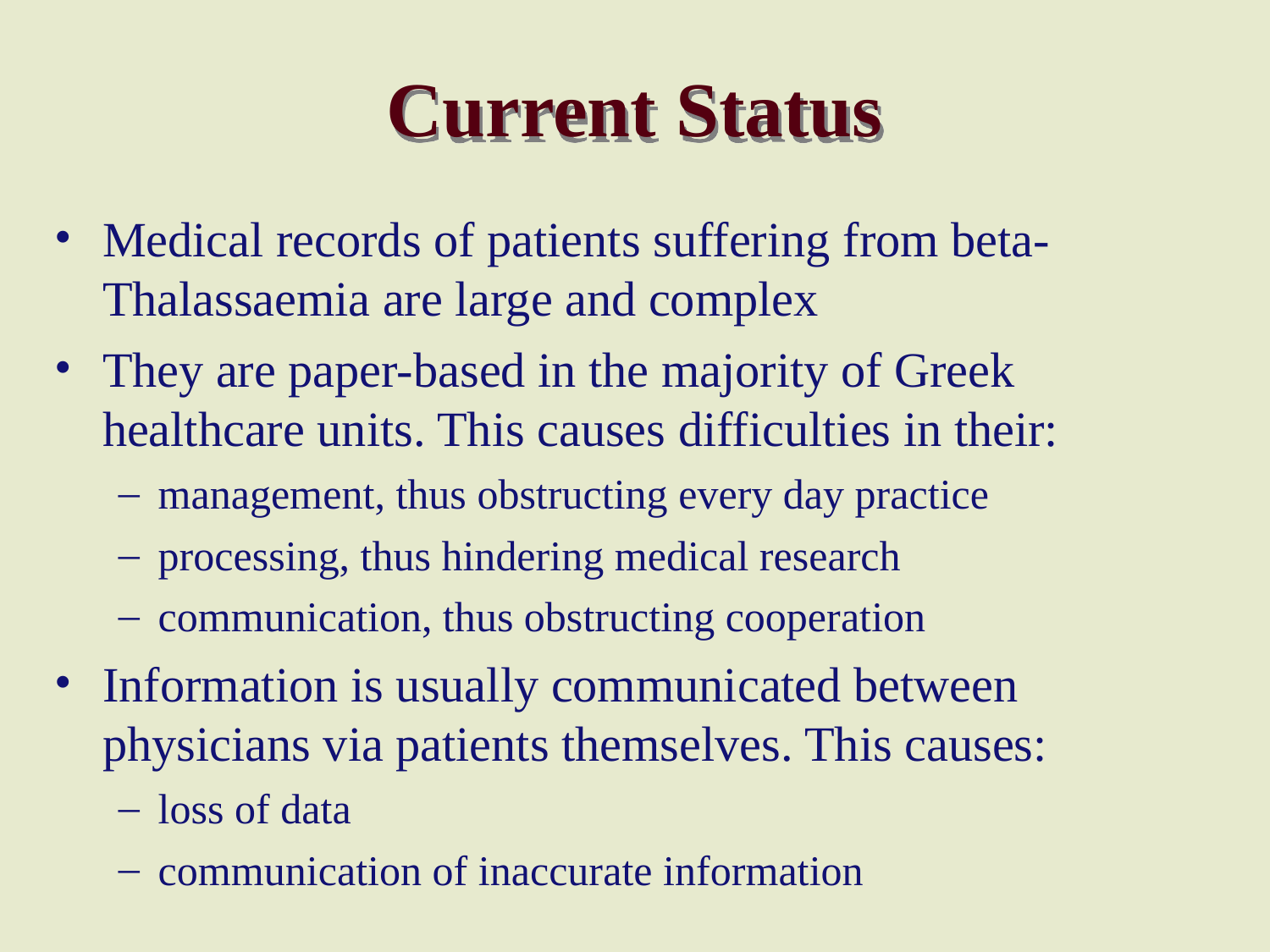

Current Status
# Medical records of patients suffering from beta-Thalassaemia are large and complex
They are paper-based in the majority of Greek healthcare units. This causes difficulties in their:
management, thus obstructing every day practice
processing, thus hindering medical research
communication, thus obstructing cooperation
Information is usually communicated between physicians via patients themselves. This causes:
loss of data
communication of inaccurate information

## Slide 5
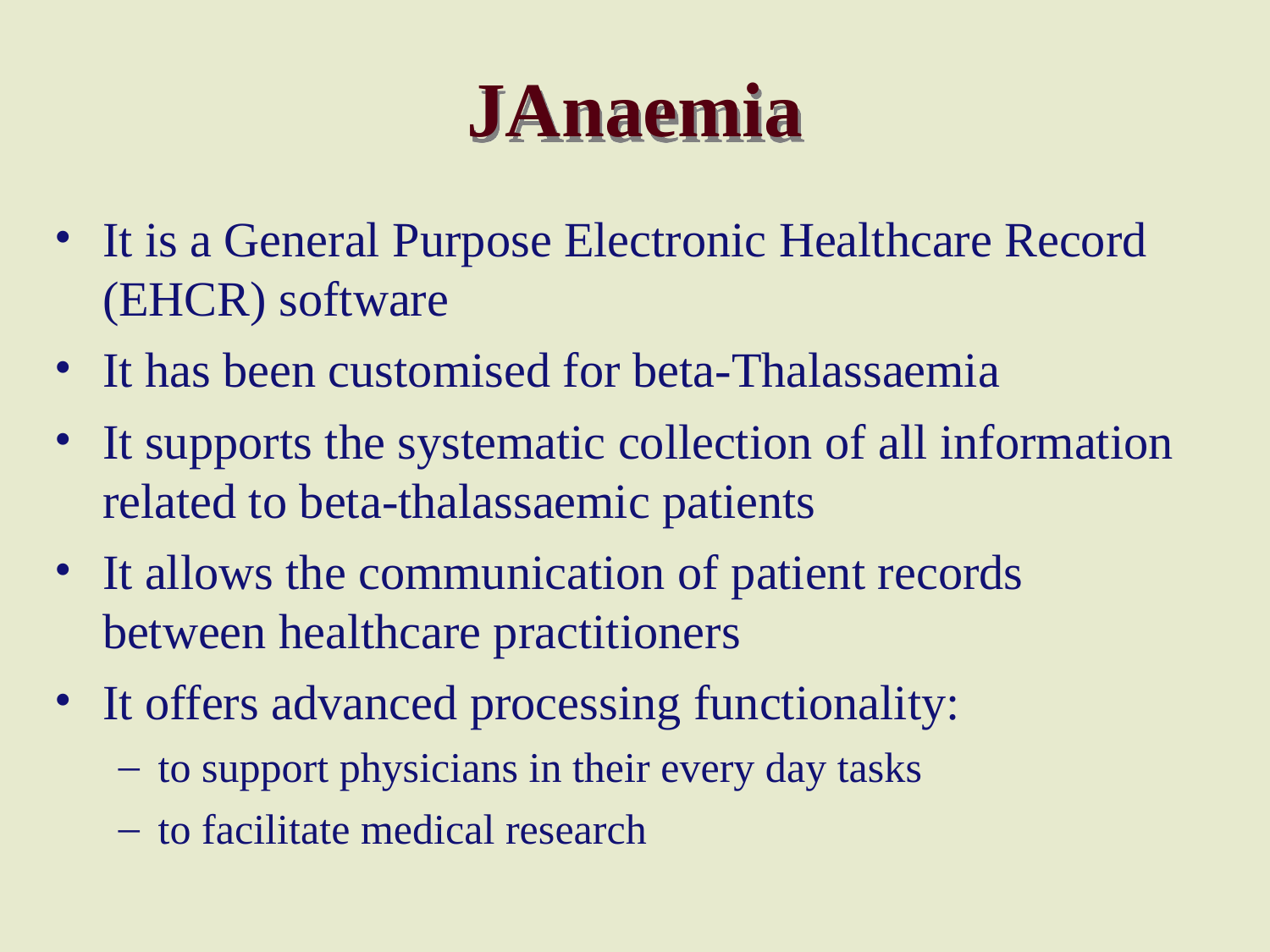

JAnaemia
# It is a General Purpose Electronic Healthcare Record (EHCR) software
It has been customised for beta-Thalassaemia
It supports the systematic collection of all information related to beta-thalassaemic patients
It allows the communication of patient records between healthcare practitioners
It offers advanced processing functionality:
to support physicians in their every day tasks
to facilitate medical research

## Slide 6
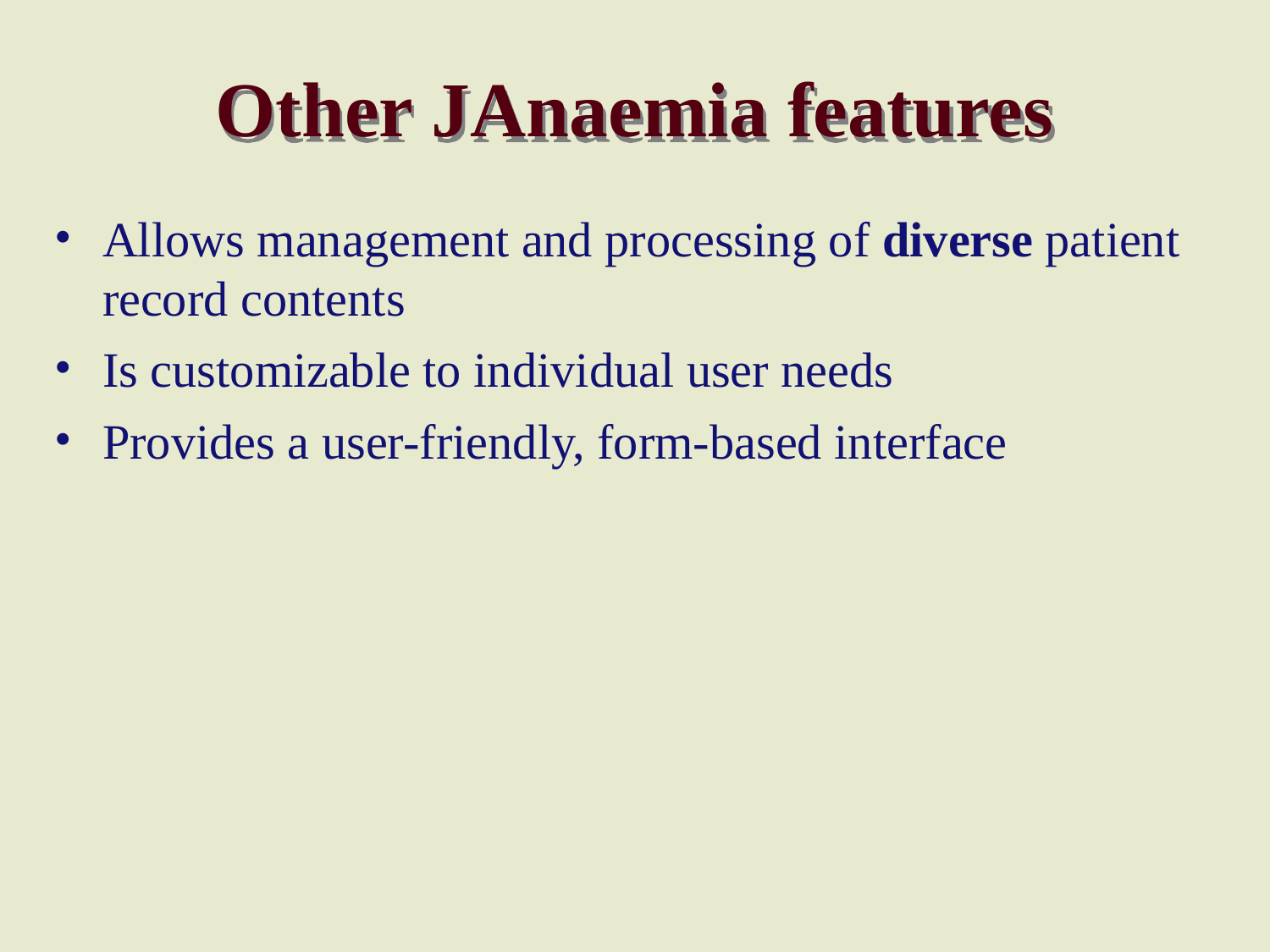

Other JAnaemia features
# Allows management and processing of diverse patient record contents
Is customizable to individual user needs
Provides a user-friendly, form-based interface

## Slide 7
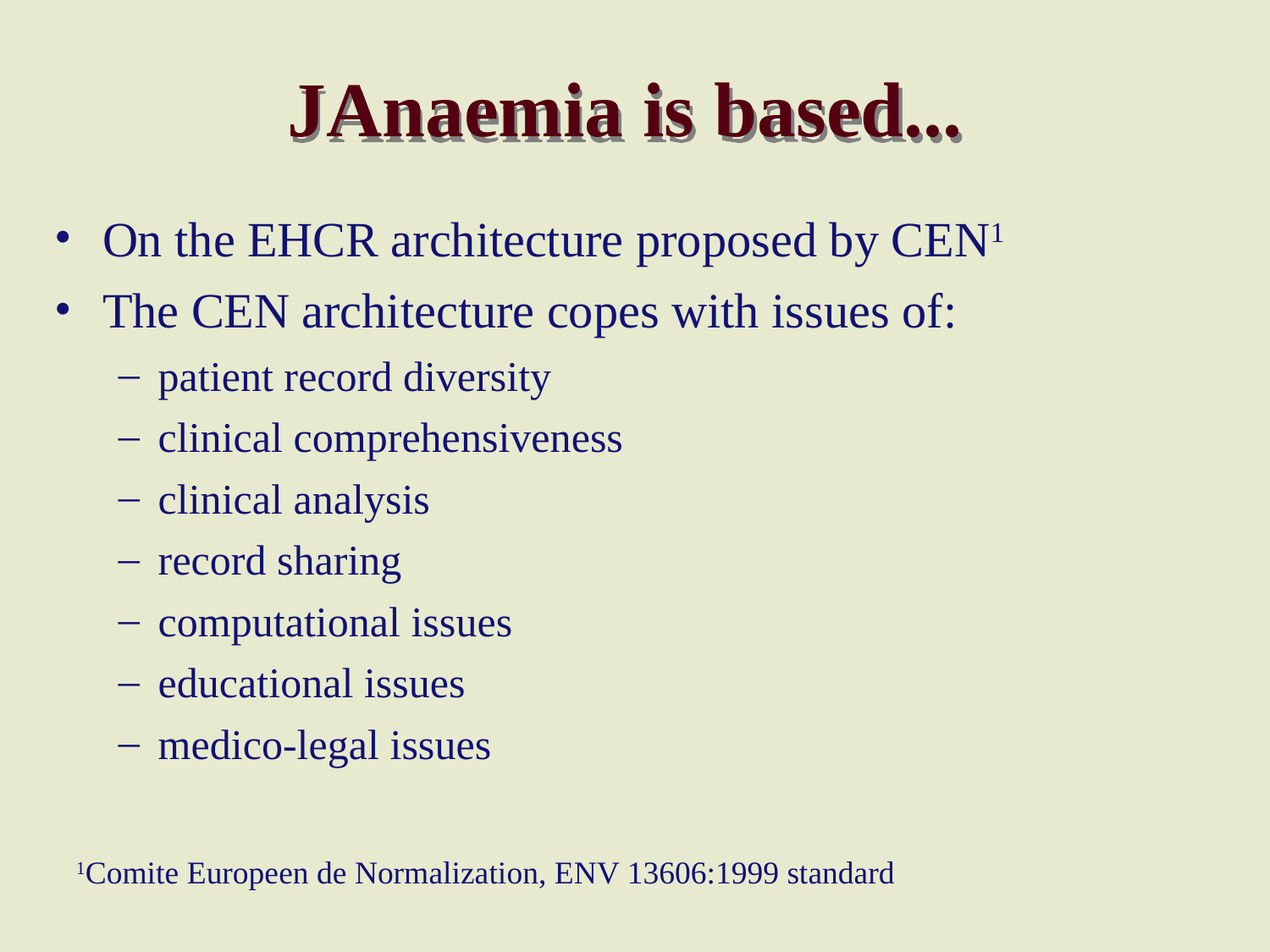

JAnaemia is based...
On the EHCR architecture proposed by CEN1
The CEN architecture copes with issues of:
patient record diversity
clinical comprehensiveness
clinical analysis
record sharing
computational issues
educational issues
medico-legal issues
1Comite Europeen de Normalization, ENV 13606:1999 standard

## Slide 8
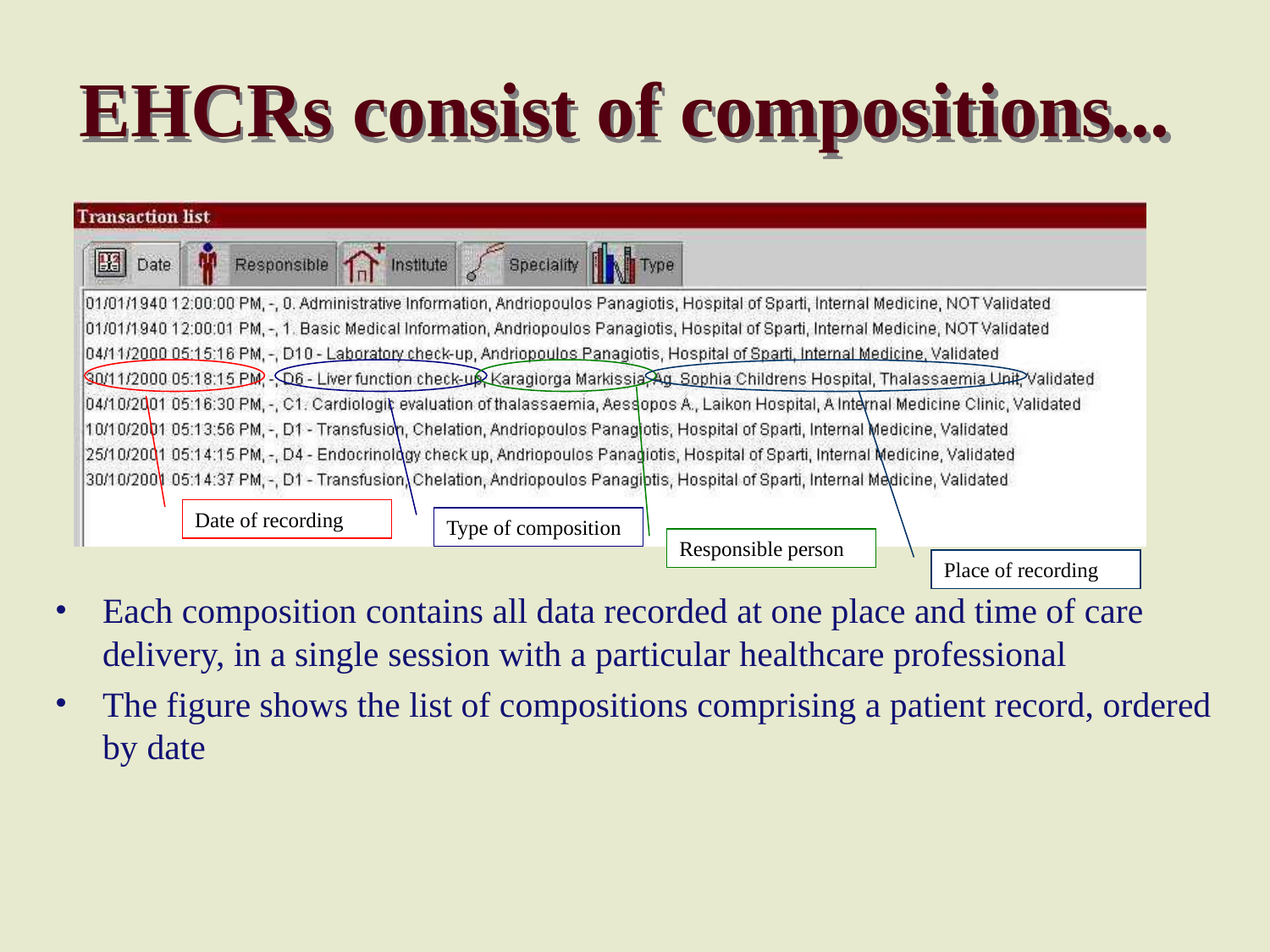

EHCRs consist of compositions...
Date of recording
Type of composition
Responsible person
Place of recording
Each composition contains all data recorded at one place and time of care delivery, in a single session with a particular healthcare professional
The figure shows the list of compositions comprising a patient record, ordered by date

## Slide 9
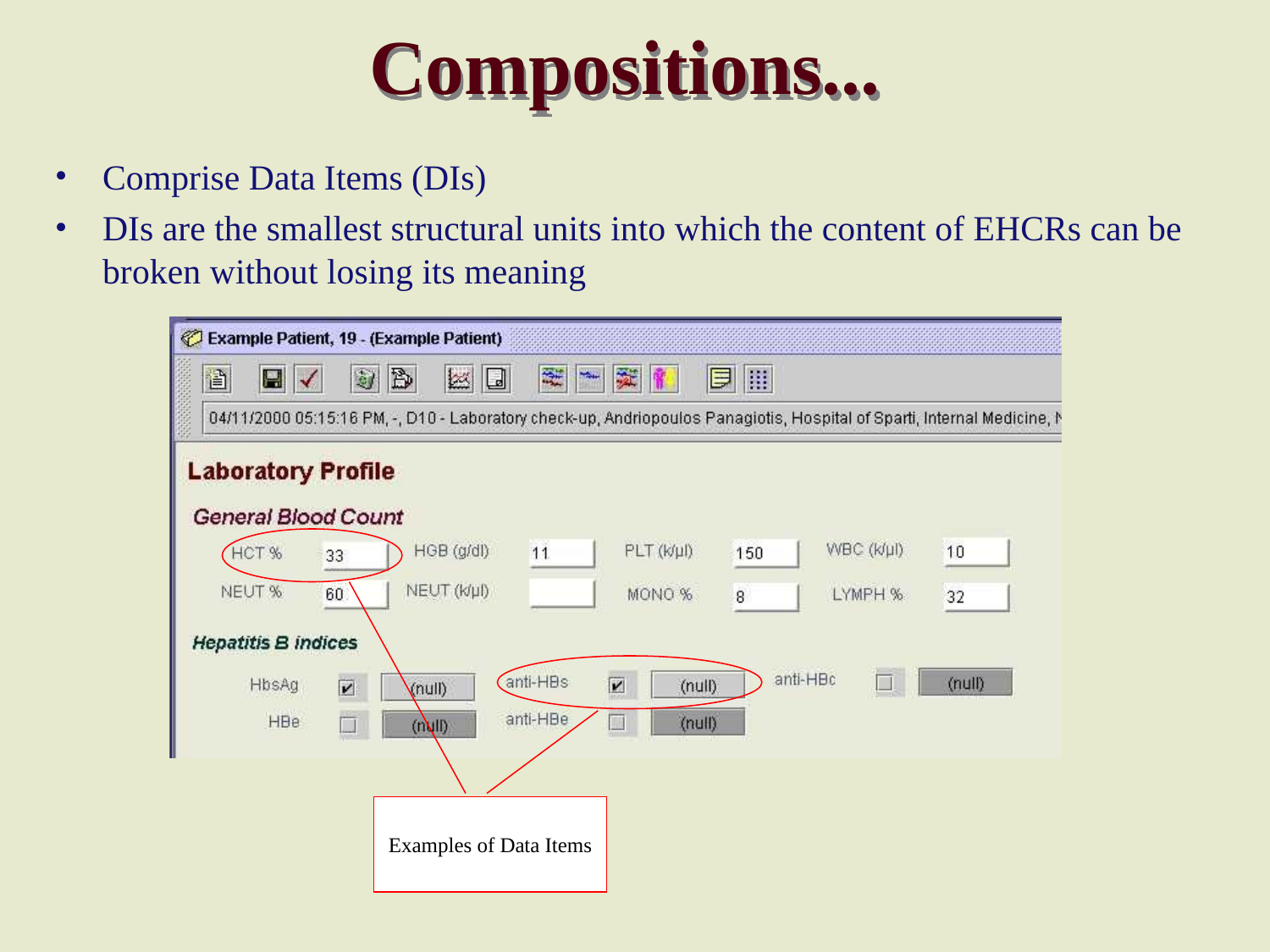

Compositions...
Comprise Data Items (DIs)
DIs are the smallest structural units into which the content of EHCRs can be broken without losing its meaning
Examples of Data Items

## Slide 10
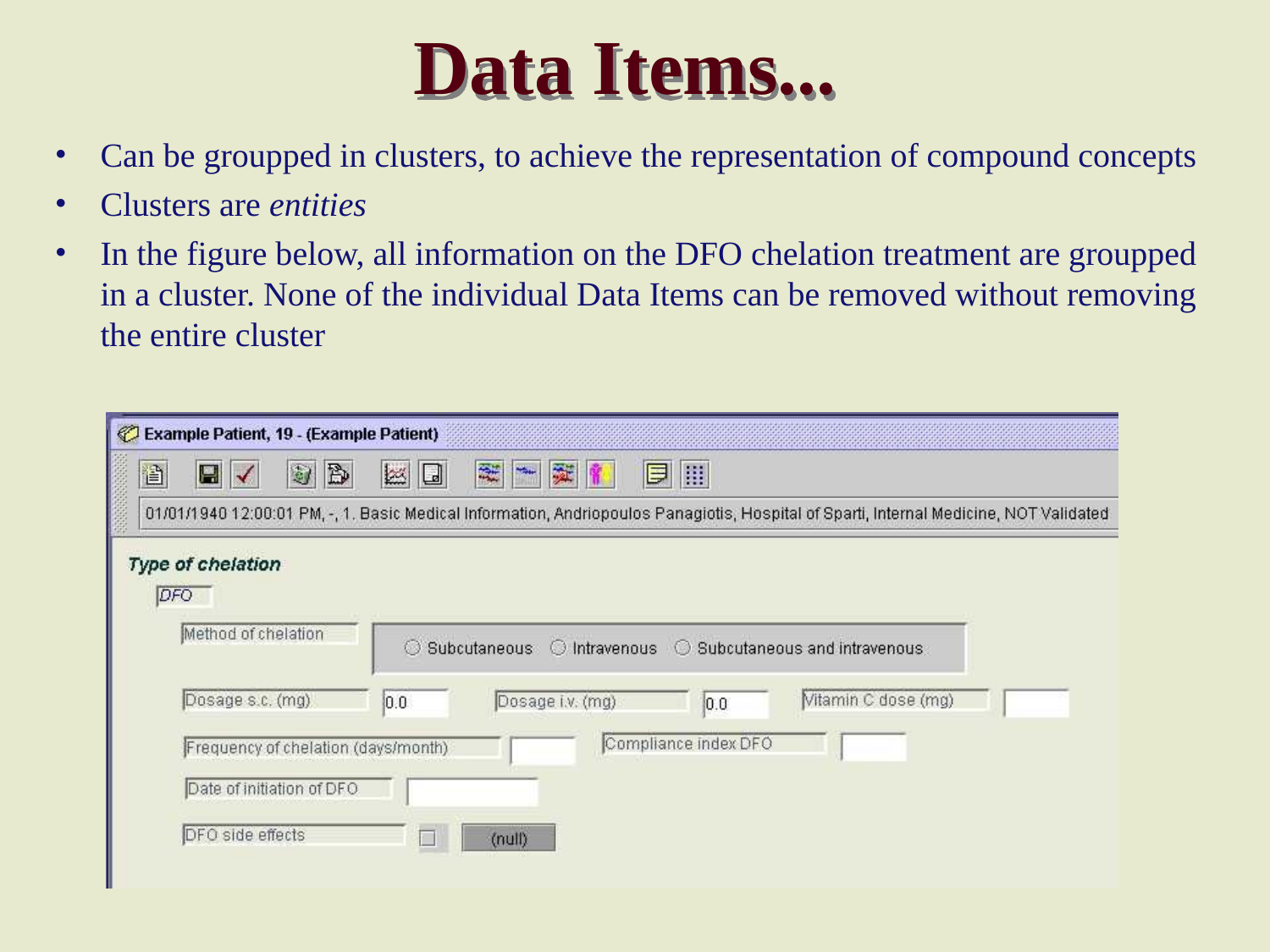

Data Items...
Can be groupped in clusters, to achieve the representation of compound concepts
Clusters are entities
In the figure below, all information on the DFO chelation treatment are groupped in a cluster. None of the individual Data Items can be removed without removing the entire cluster

## Slide 11
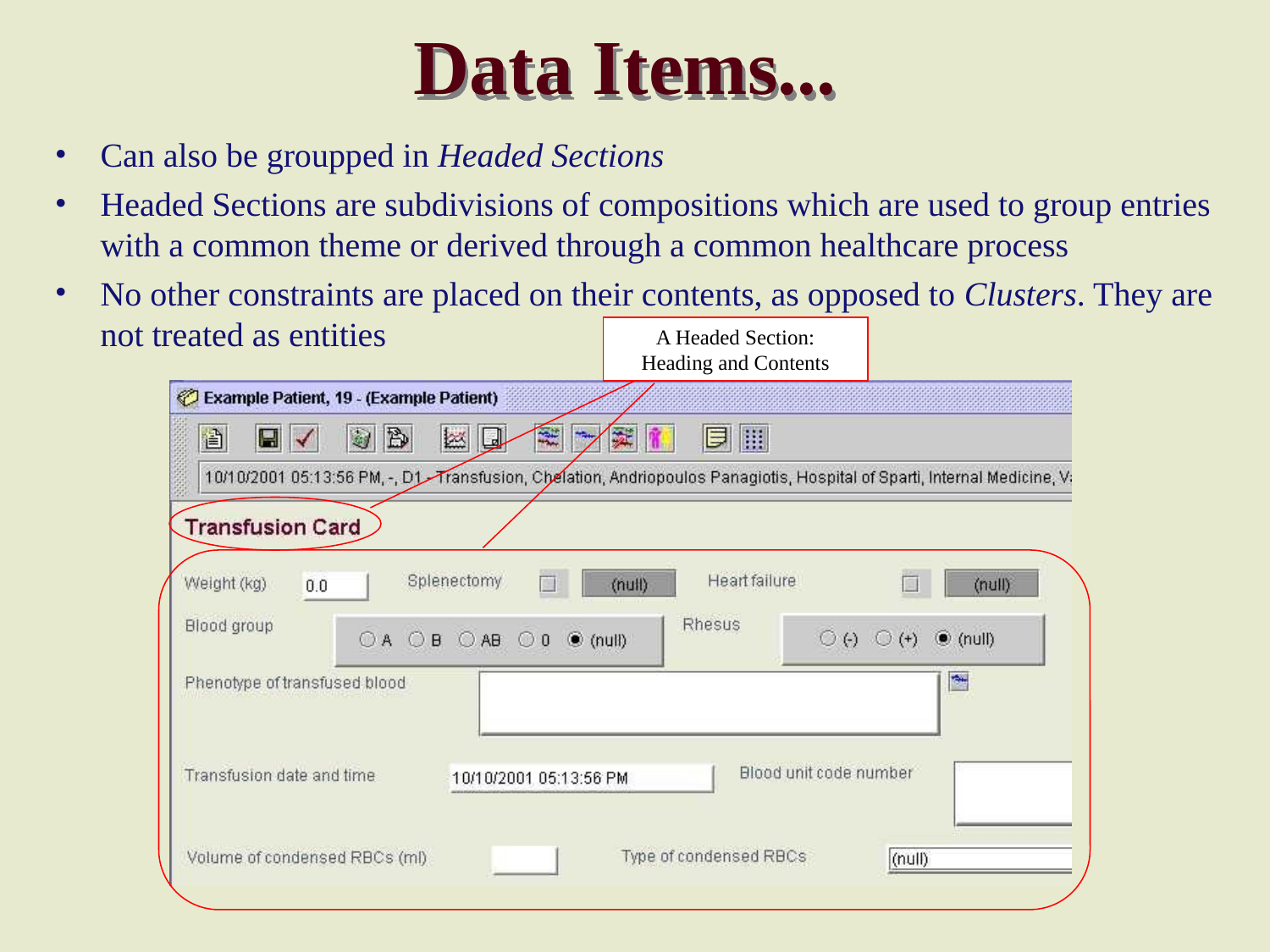

Data Items...
Can also be groupped in Headed Sections
Headed Sections are subdivisions of compositions which are used to group entries with a common theme or derived through a common healthcare process
No other constraints are placed on their contents, as opposed to Clusters. They are not treated as entities
A Headed Section:
Heading and Contents

## Slide 12
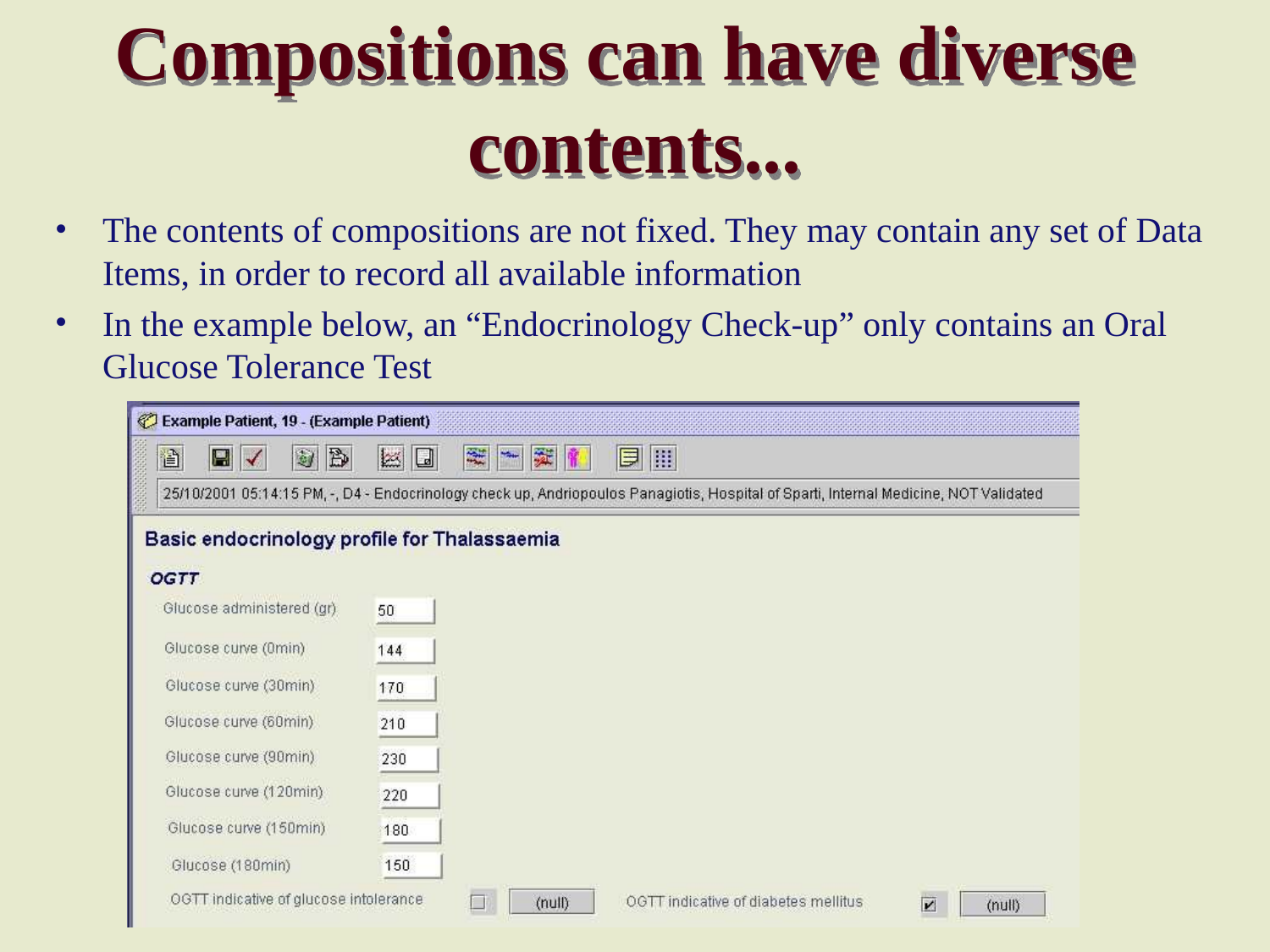

Compositions can have diverse contents...
The contents of compositions are not fixed. They may contain any set of Data Items, in order to record all available information
In the example below, an “Endocrinology Check-up” only contains an Oral Glucose Tolerance Test

## Slide 13
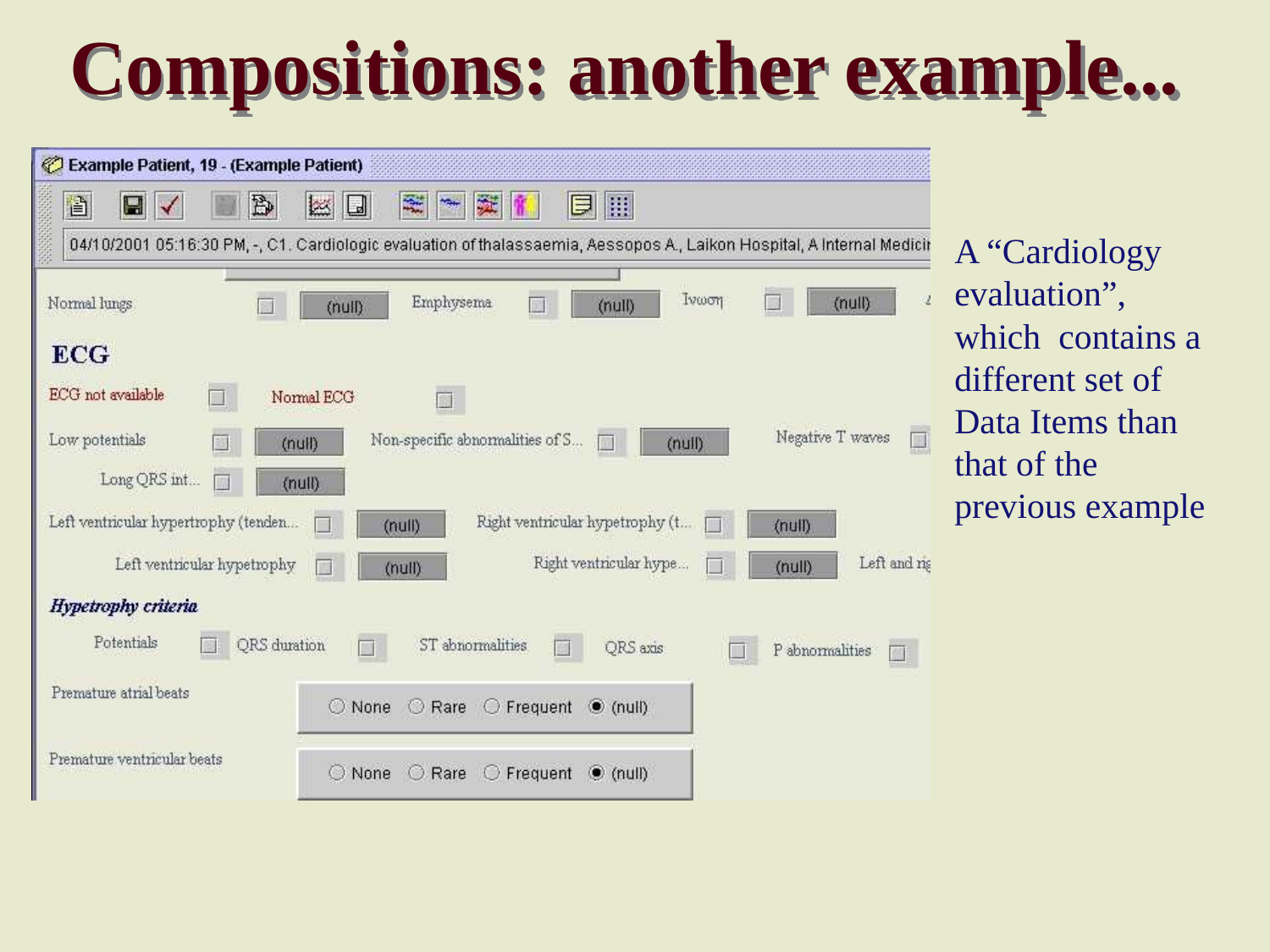

Compositions: another example...
A “Cardiology evaluation”, which contains a different set of Data Items than that of the previous example

## Slide 14
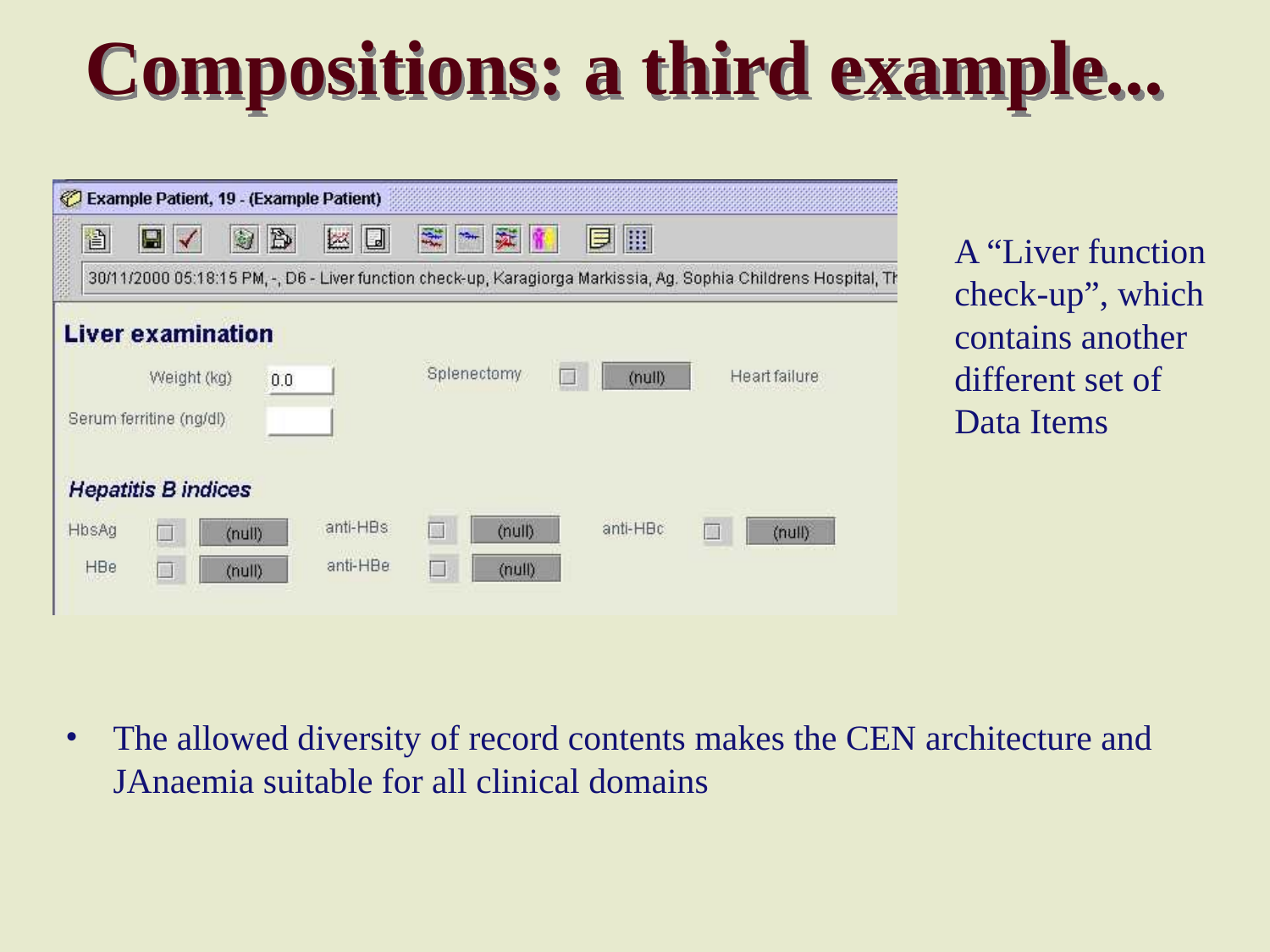

Compositions: a third example...
A “Liver function check-up”, which contains another different set of Data Items
The allowed diversity of record contents makes the CEN architecture and JAnaemia suitable for all clinical domains

## Slide 15
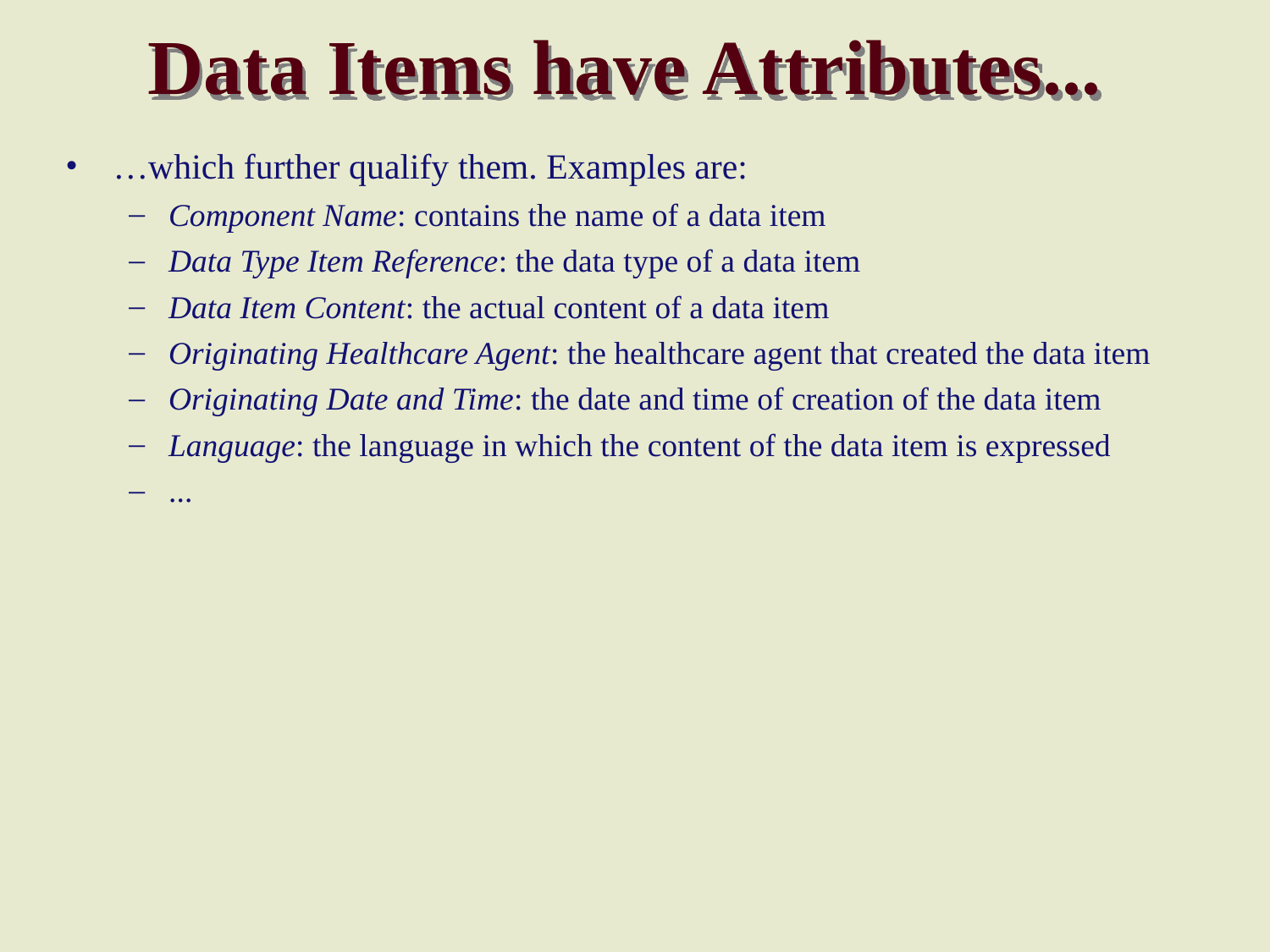

Data Items have Attributes...
…which further qualify them. Examples are:
Component Name: contains the name of a data item
Data Type Item Reference: the data type of a data item
Data Item Content: the actual content of a data item
Originating Healthcare Agent: the healthcare agent that created the data item
Originating Date and Time: the date and time of creation of the data item
Language: the language in which the content of the data item is expressed
...

## Slide 16
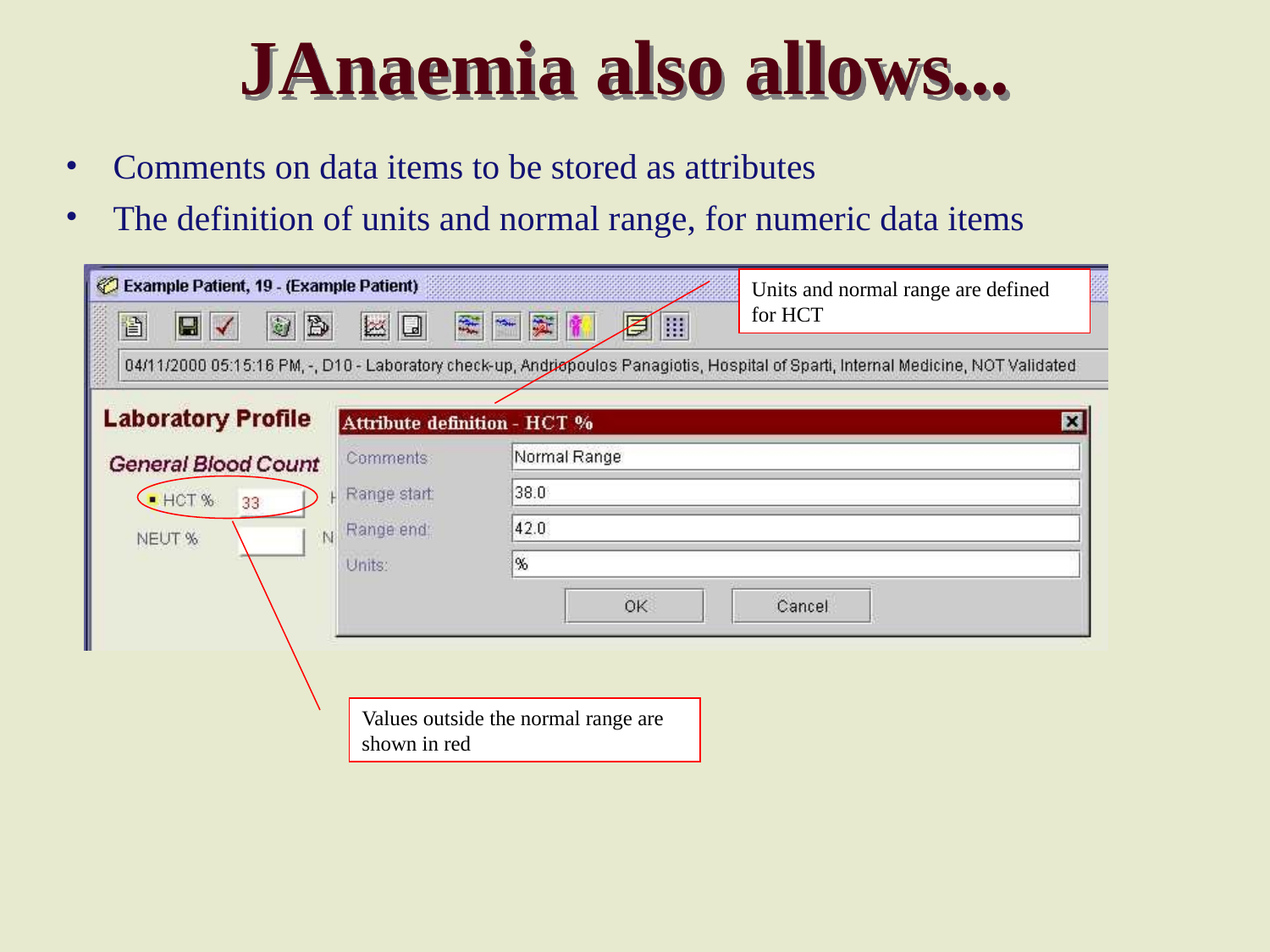

JAnaemia also allows...
Comments on data items to be stored as attributes
The definition of units and normal range, for numeric data items
Units and normal range are defined for HCT
Values outside the normal range are shown in red

## Slide 17
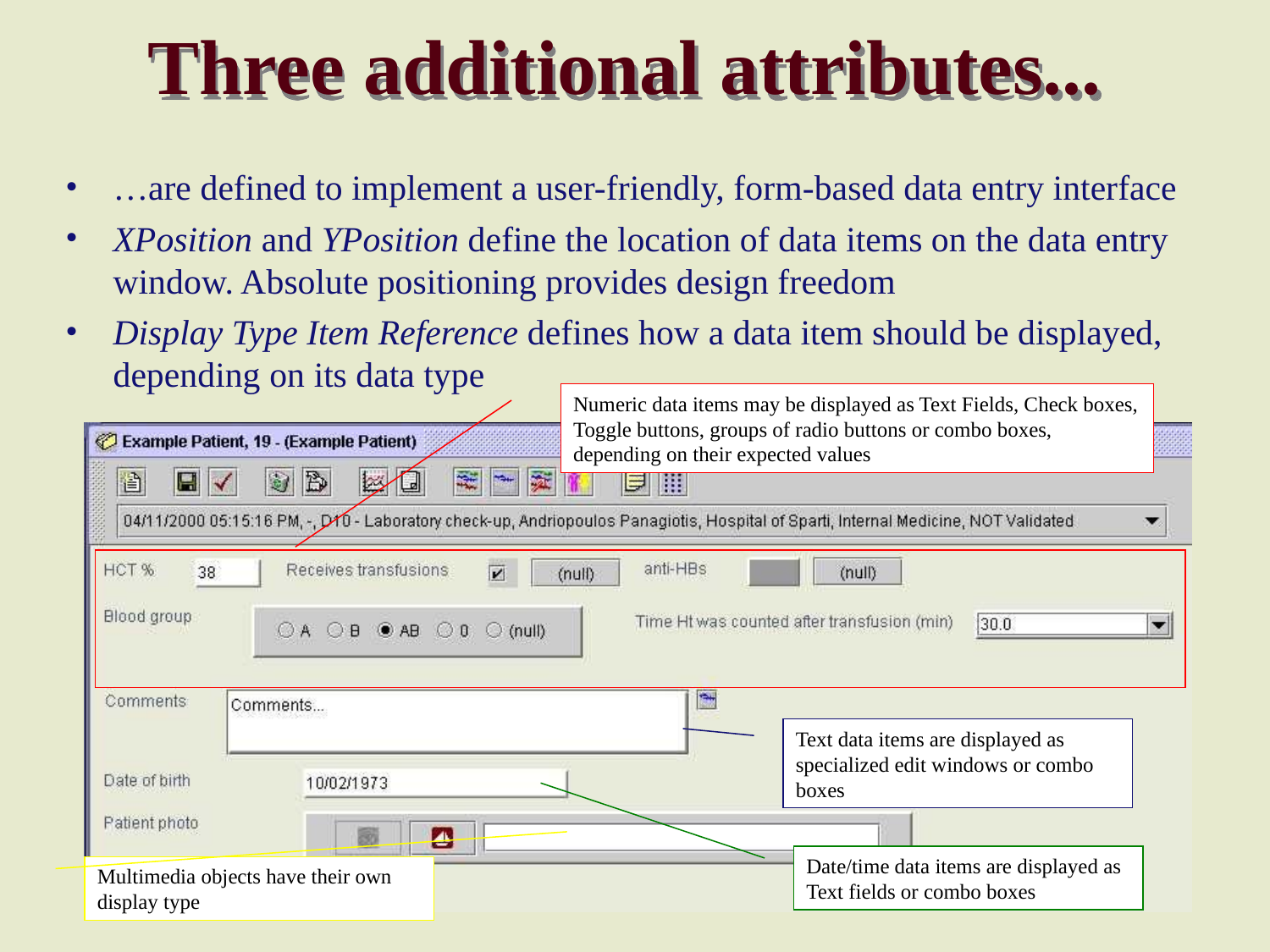

Three additional attributes...
…are defined to implement a user-friendly, form-based data entry interface
XPosition and YPosition define the location of data items on the data entry window. Absolute positioning provides design freedom
Display Type Item Reference defines how a data item should be displayed, depending on its data type
Numeric data items may be displayed as Text Fields, Check boxes, Toggle buttons, groups of radio buttons or combo boxes, depending on their expected values
Text data items are displayed as specialized edit windows or combo boxes
Date/time data items are displayed as Text fields or combo boxes
Multimedia objects have their own display type

## Slide 18
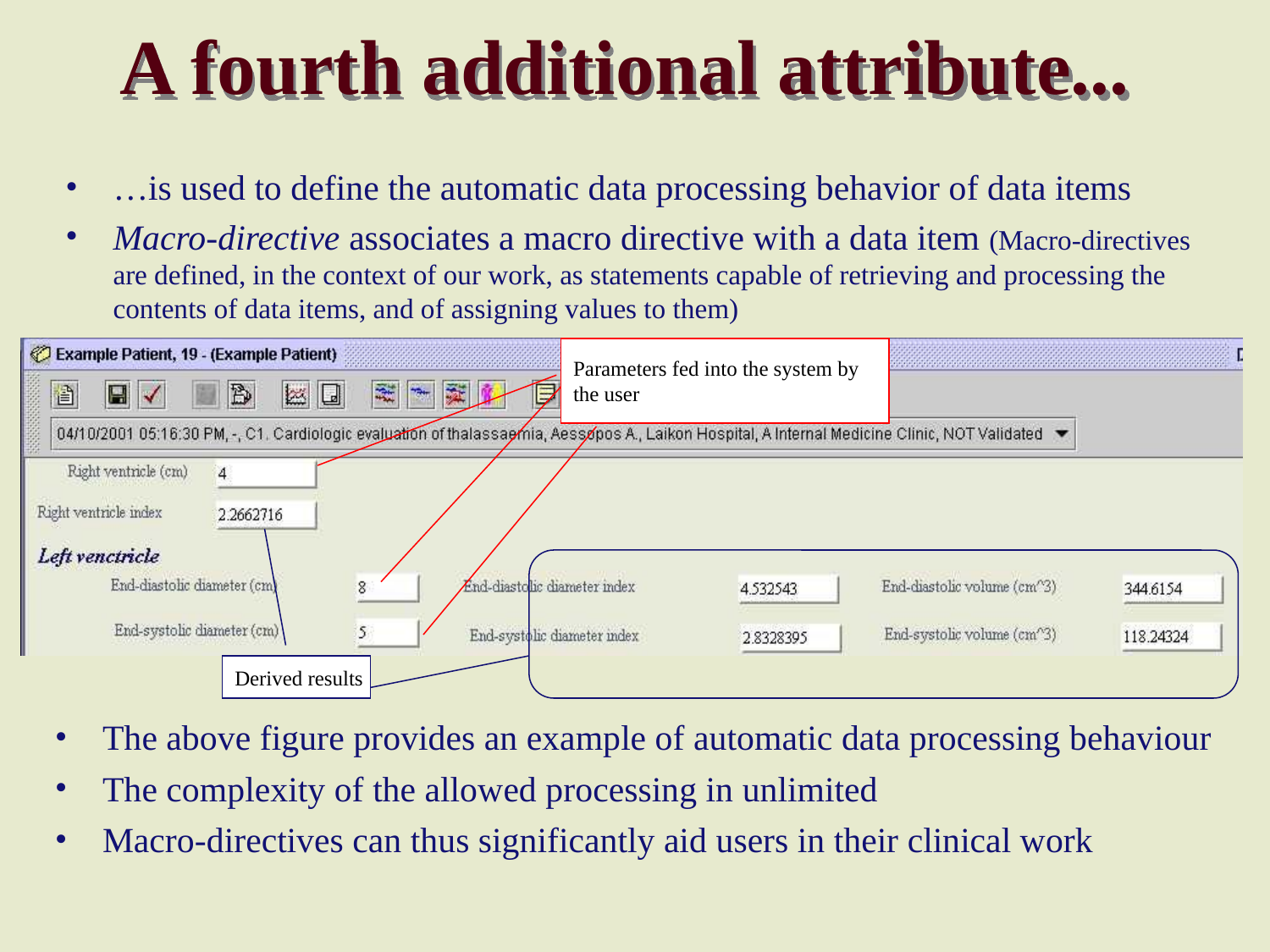

A fourth additional attribute...
…is used to define the automatic data processing behavior of data items
Macro-directive associates a macro directive with a data item (Macro-directives are defined, in the context of our work, as statements capable of retrieving and processing the contents of data items, and of assigning values to them)
Parameters fed into the system by
the user
Derived results
The above figure provides an example of automatic data processing behaviour
The complexity of the allowed processing in unlimited
Macro-directives can thus significantly aid users in their clinical work

## Slide 19
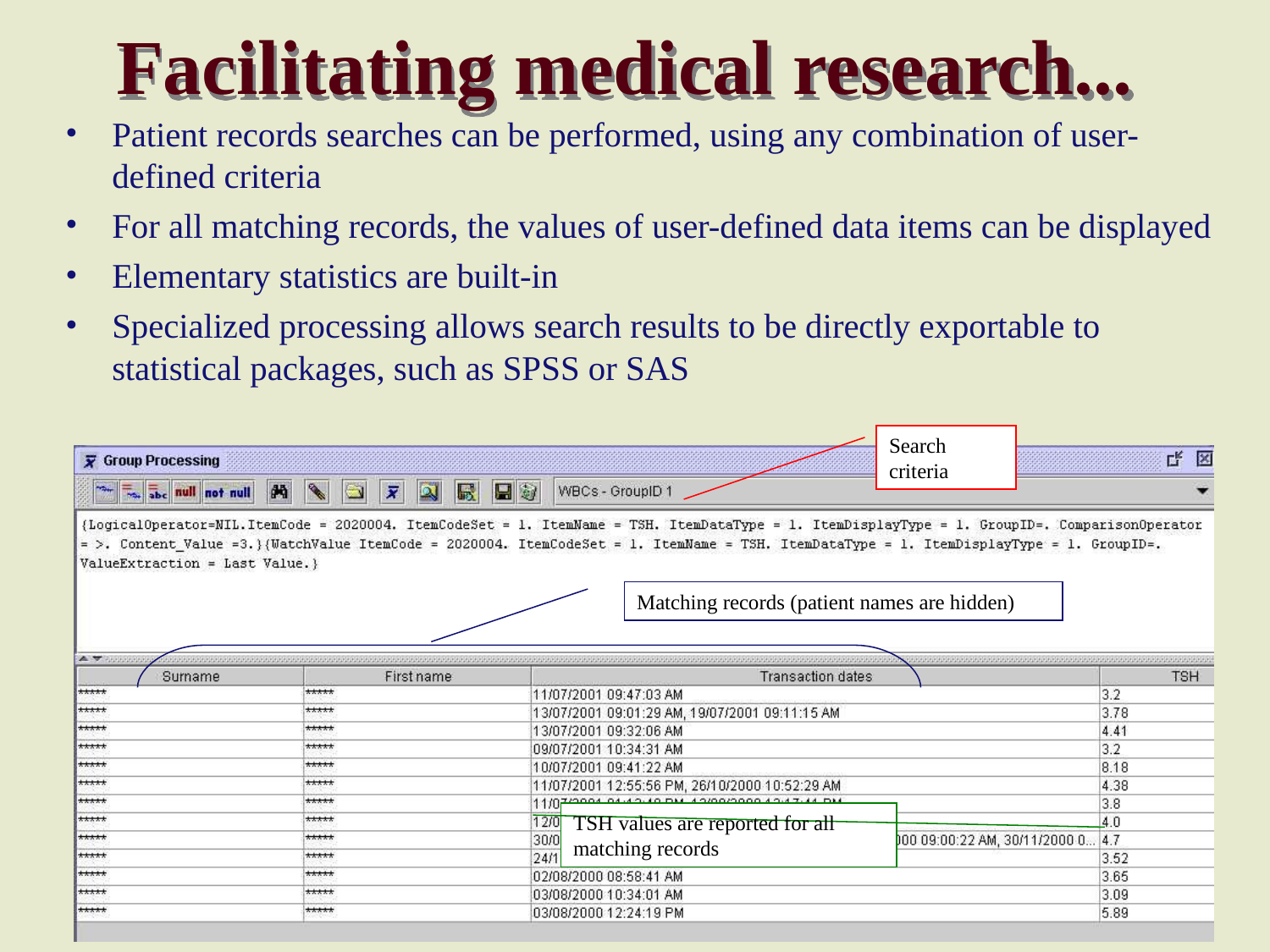

Facilitating medical research...
Patient records searches can be performed, using any combination of user-defined criteria
For all matching records, the values of user-defined data items can be displayed
Elementary statistics are built-in
Specialized processing allows search results to be directly exportable to statistical packages, such as SPSS or SAS
Search criteria
Matching records (patient names are hidden)
TSH values are reported for all matching records

## Slide 20
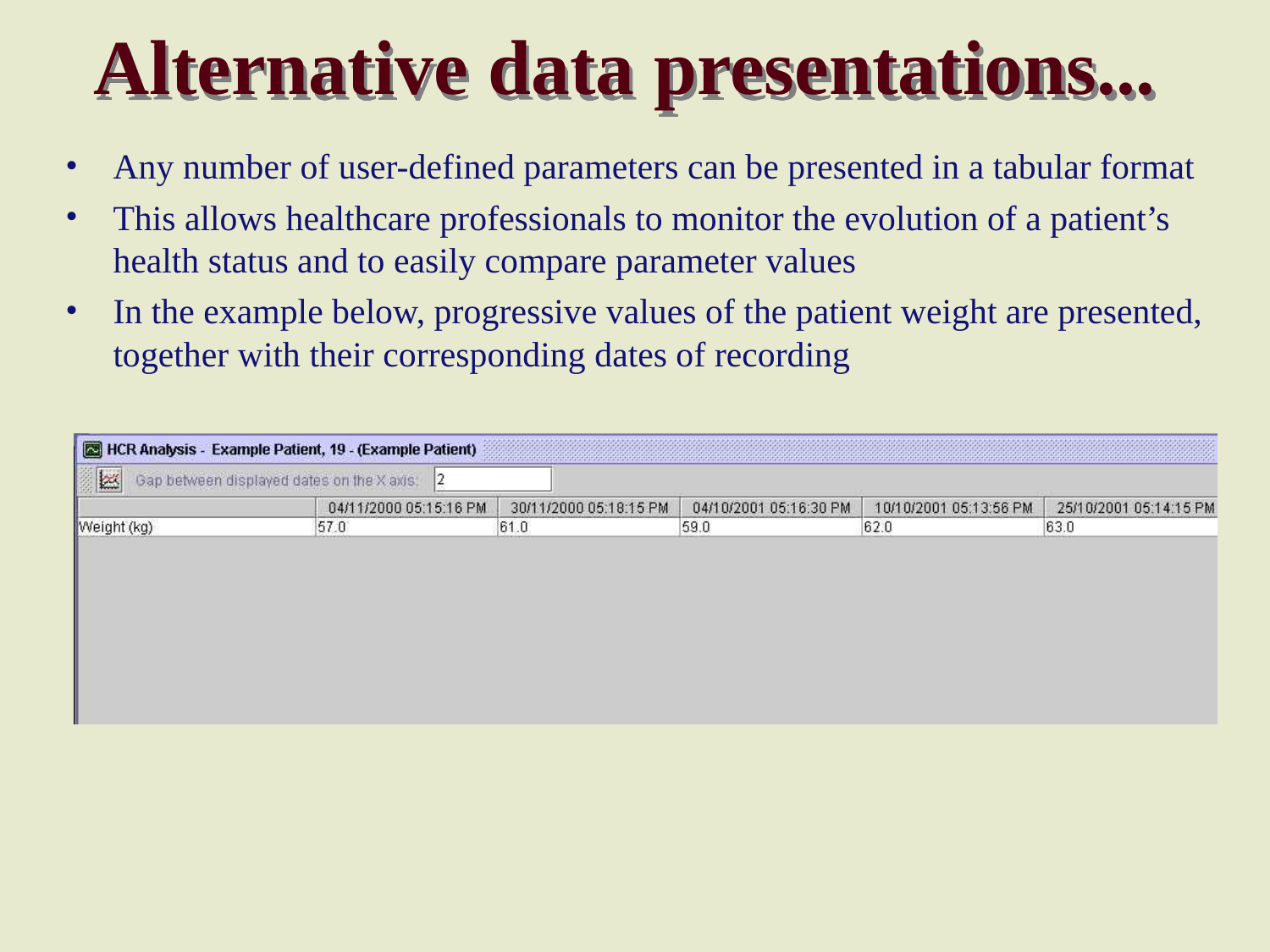

Alternative data presentations...
Any number of user-defined parameters can be presented in a tabular format
This allows healthcare professionals to monitor the evolution of a patient’s health status and to easily compare parameter values
In the example below, progressive values of the patient weight are presented, together with their corresponding dates of recording

## Slide 21
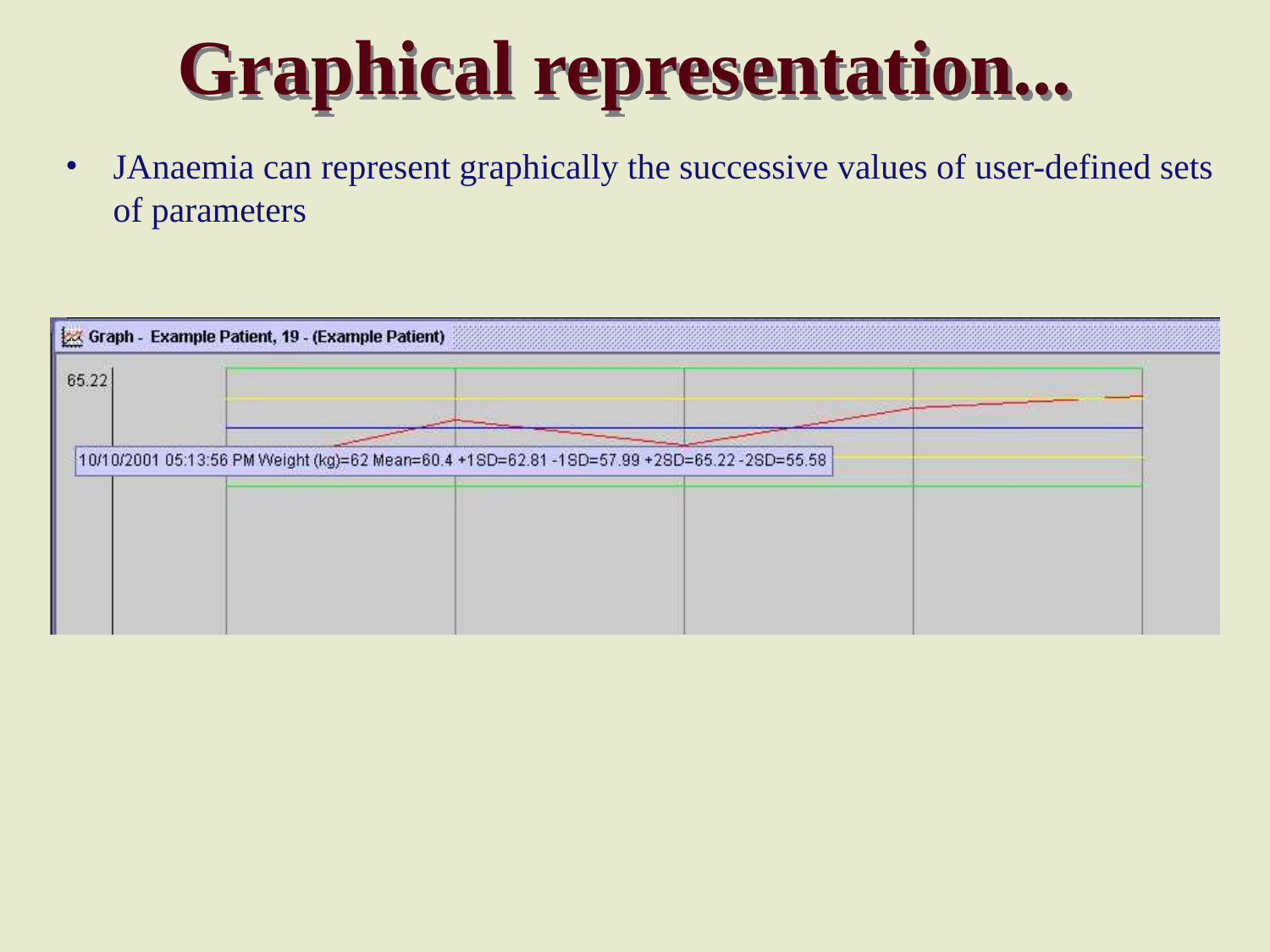

Graphical representation...
JAnaemia can represent graphically the successive values of user-defined sets of parameters

## Slide 22
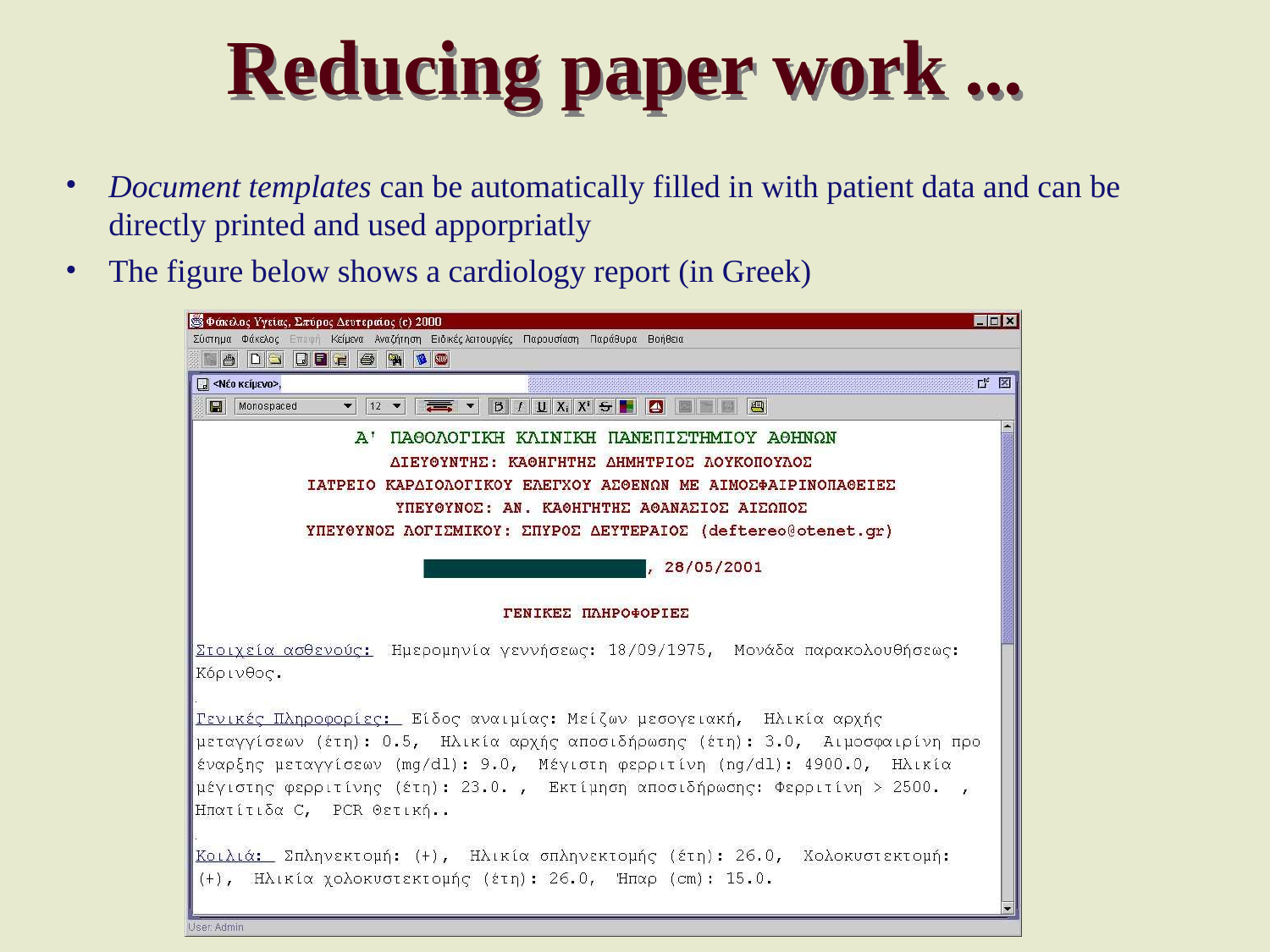

Reducing paper work ...
Document templates can be automatically filled in with patient data and can be directly printed and used apporpriatly
The figure below shows a cardiology report (in Greek)

## Slide 23
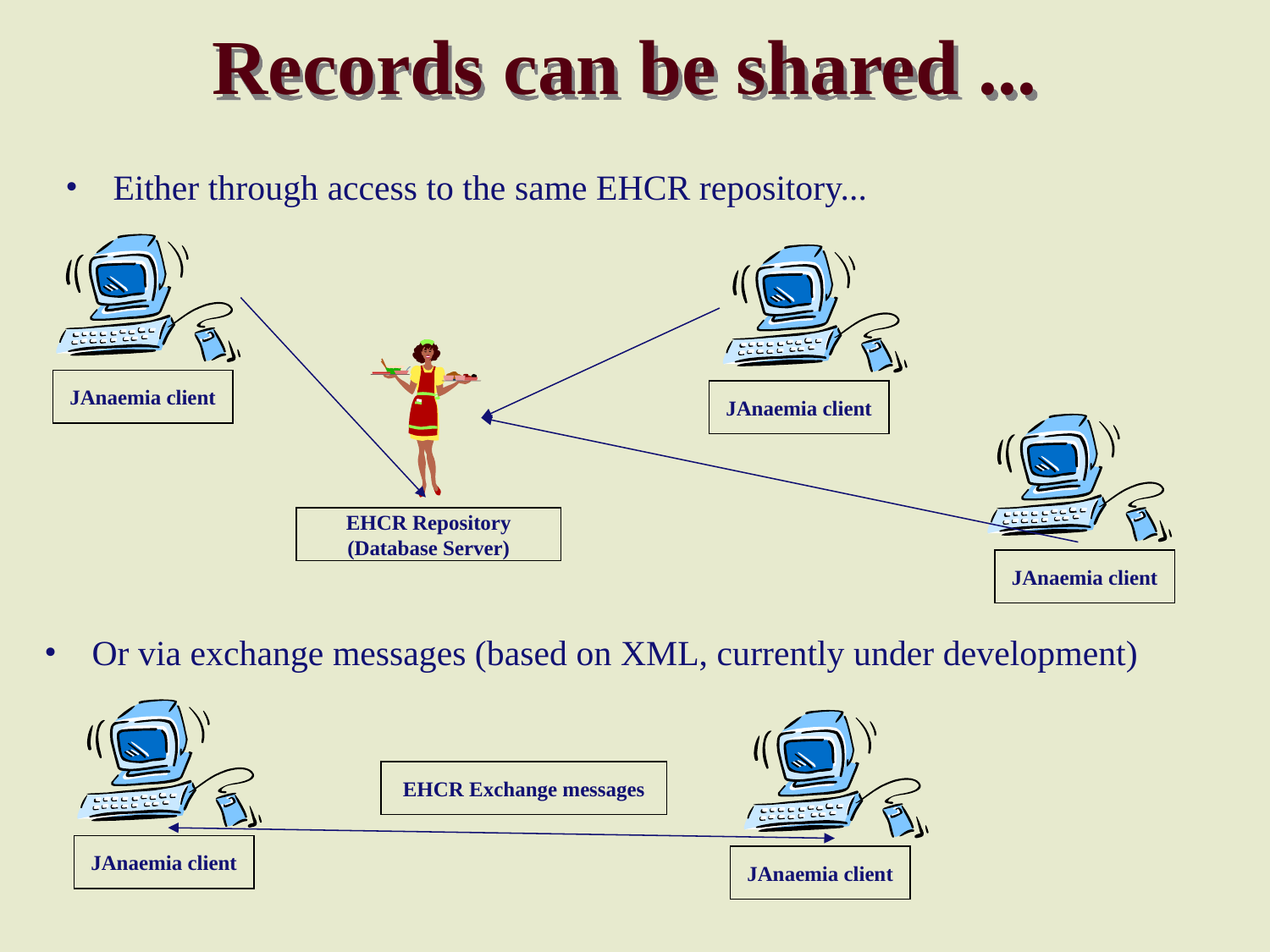

Records can be shared ...
Either through access to the same EHCR repository...
JAnaemia client
JAnaemia client
EHCR Repository
(Database Server)
JAnaemia client
Or via exchange messages (based on XML, currently under development)
EHCR Exchange messages
JAnaemia client
JAnaemia client

## Slide 24
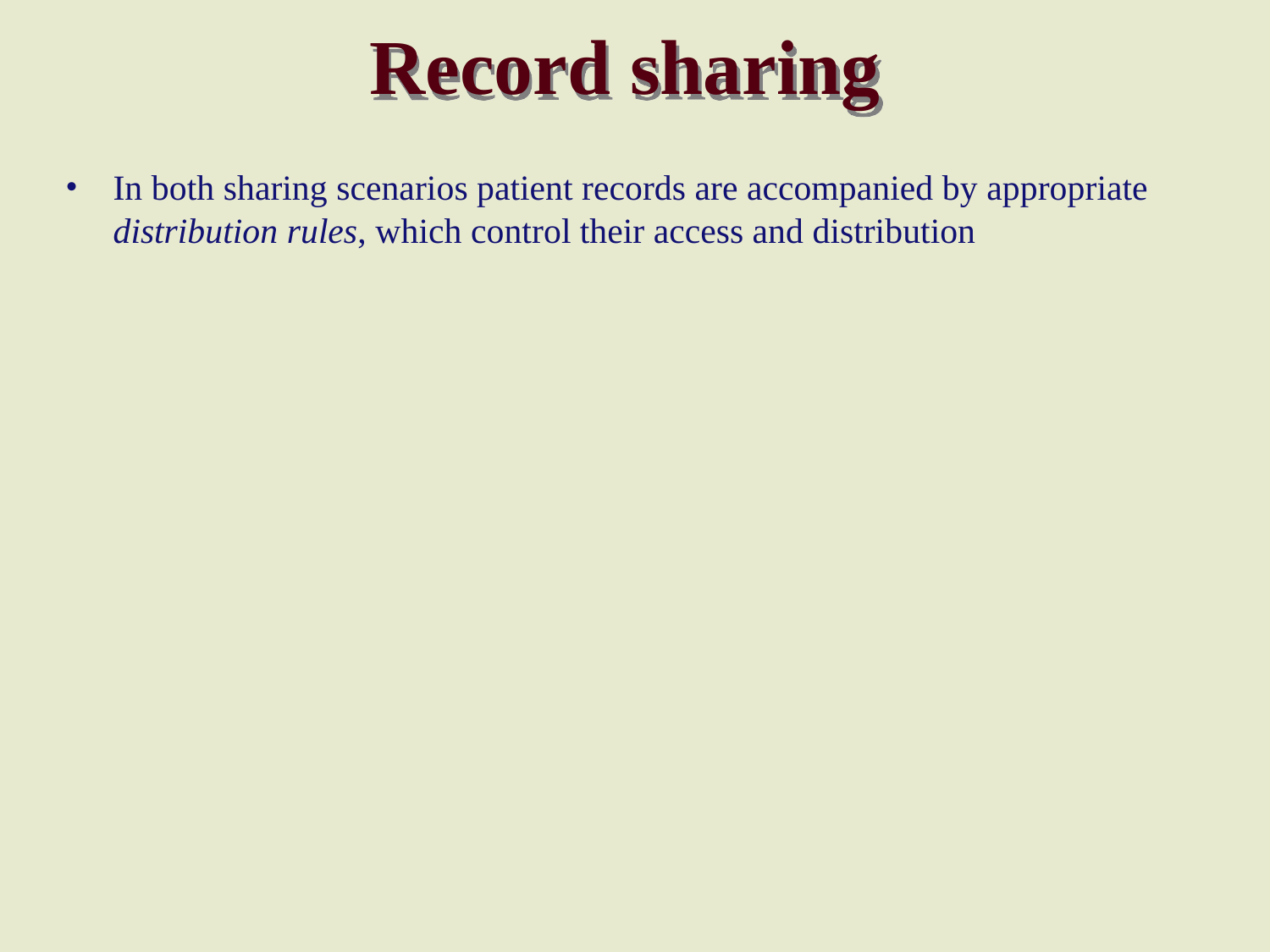

Record sharing
In both sharing scenarios patient records are accompanied by appropriate distribution rules, which control their access and distribution

## Slide 25
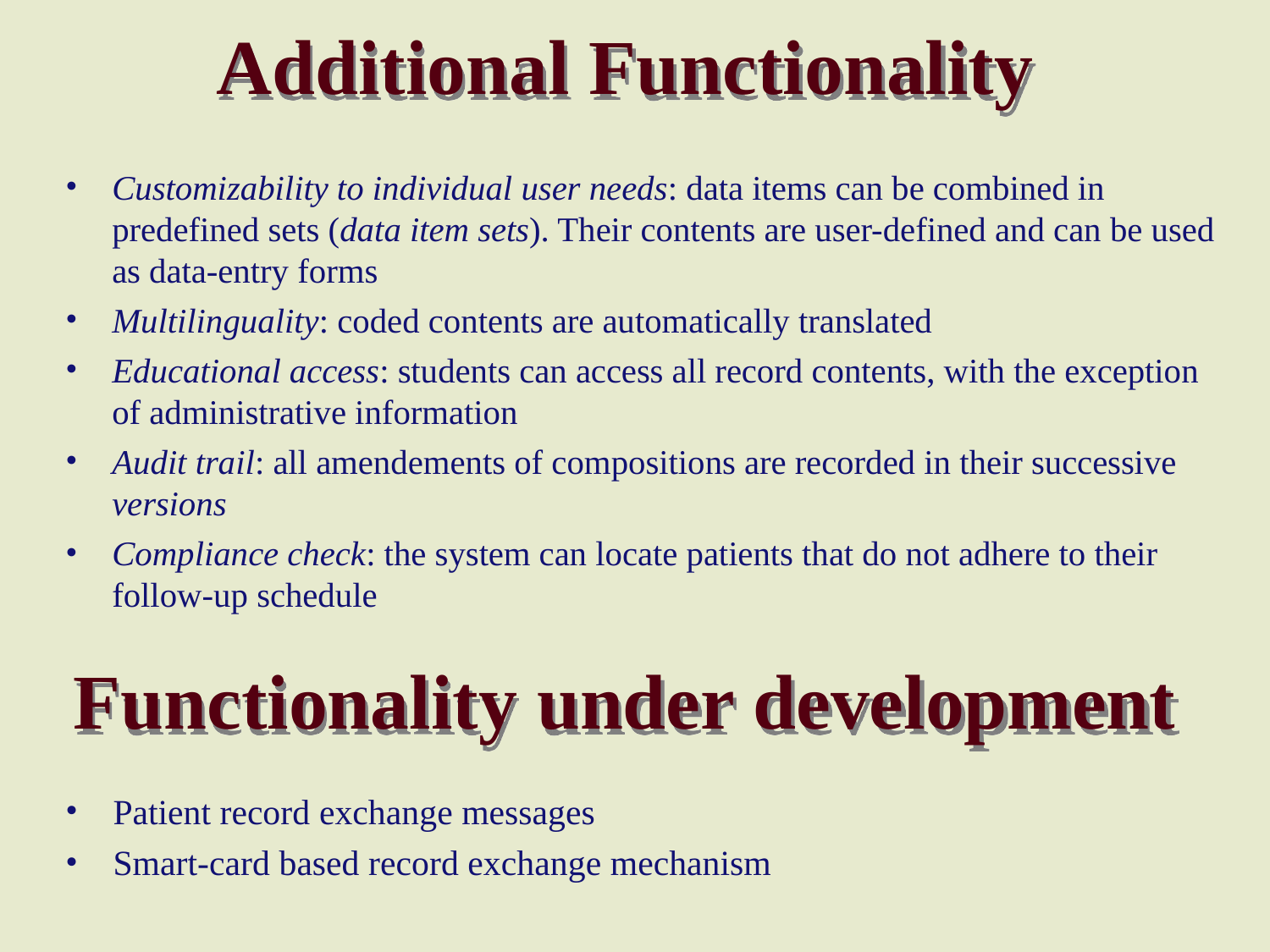

Additional Functionality
Customizability to individual user needs: data items can be combined in predefined sets (data item sets). Their contents are user-defined and can be used as data-entry forms
Multilinguality: coded contents are automatically translated
Educational access: students can access all record contents, with the exception of administrative information
Audit trail: all amendements of compositions are recorded in their successive versions
Compliance check: the system can locate patients that do not adhere to their follow-up schedule
Functionality under development
Patient record exchange messages
Smart-card based record exchange mechanism

## Slide 26
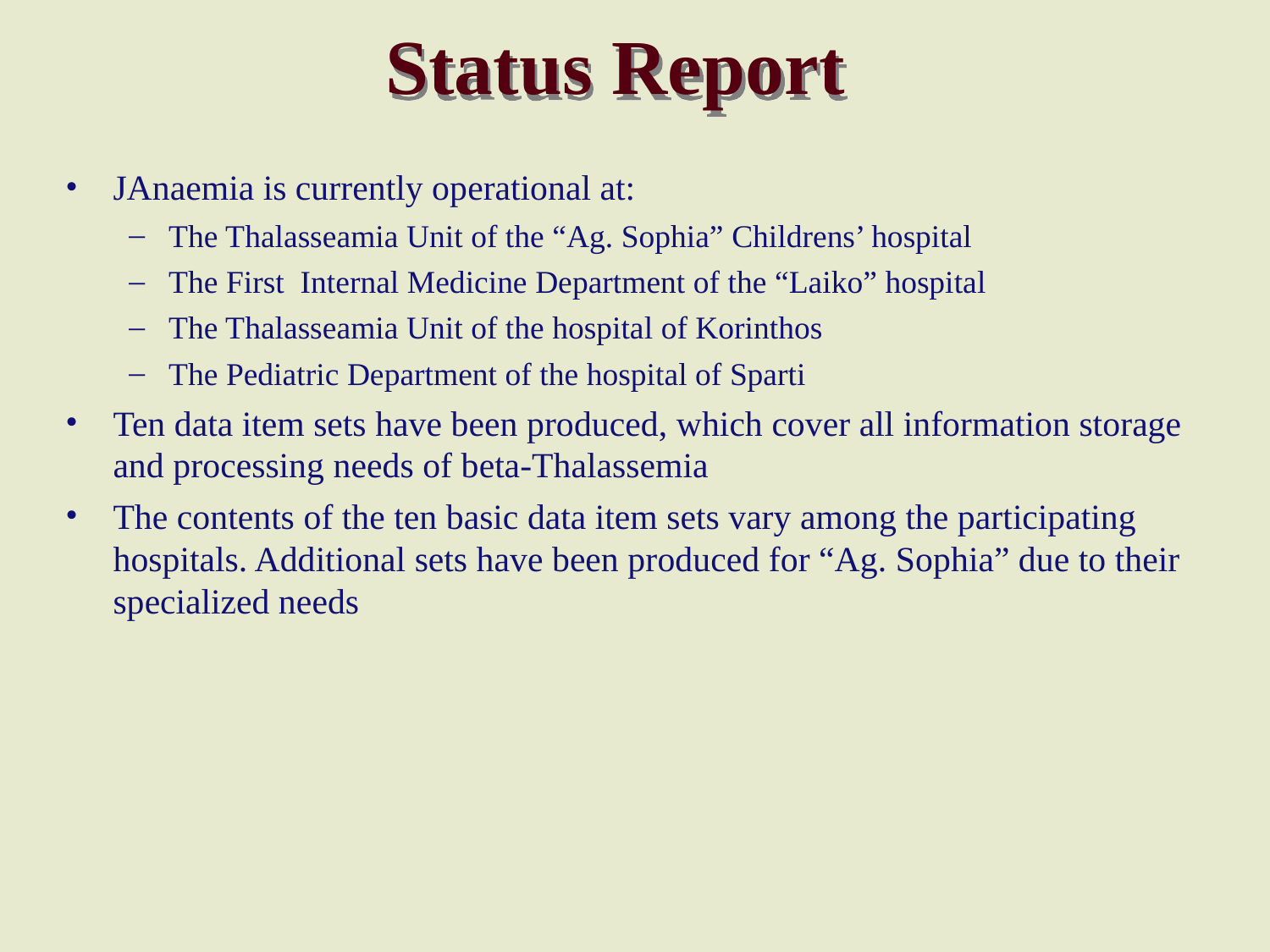

Status Report
JAnaemia is currently operational at:
The Thalasseamia Unit of the “Ag. Sophia” Childrens’ hospital
The First Internal Medicine Department of the “Laiko” hospital
The Thalasseamia Unit of the hospital of Korinthos
The Pediatric Department of the hospital of Sparti
Ten data item sets have been produced, which cover all information storage and processing needs of beta-Thalassemia
The contents of the ten basic data item sets vary among the participating hospitals. Additional sets have been produced for “Ag. Sophia” due to their specialized needs

## Slide 27
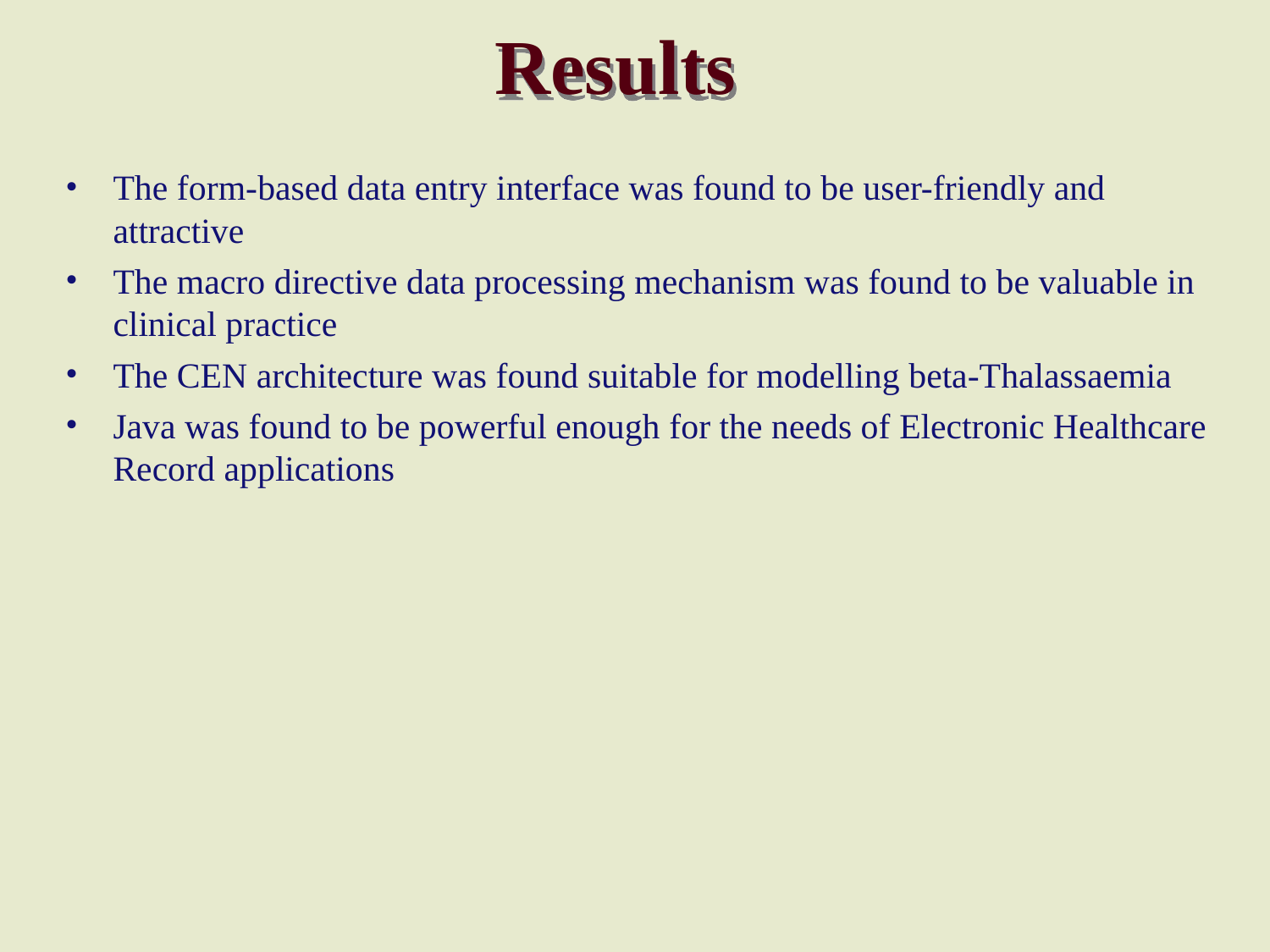

Results
The form-based data entry interface was found to be user-friendly and attractive
The macro directive data processing mechanism was found to be valuable in clinical practice
The CEN architecture was found suitable for modelling beta-Thalassaemia
Java was found to be powerful enough for the needs of Electronic Healthcare Record applications

## Slide 28
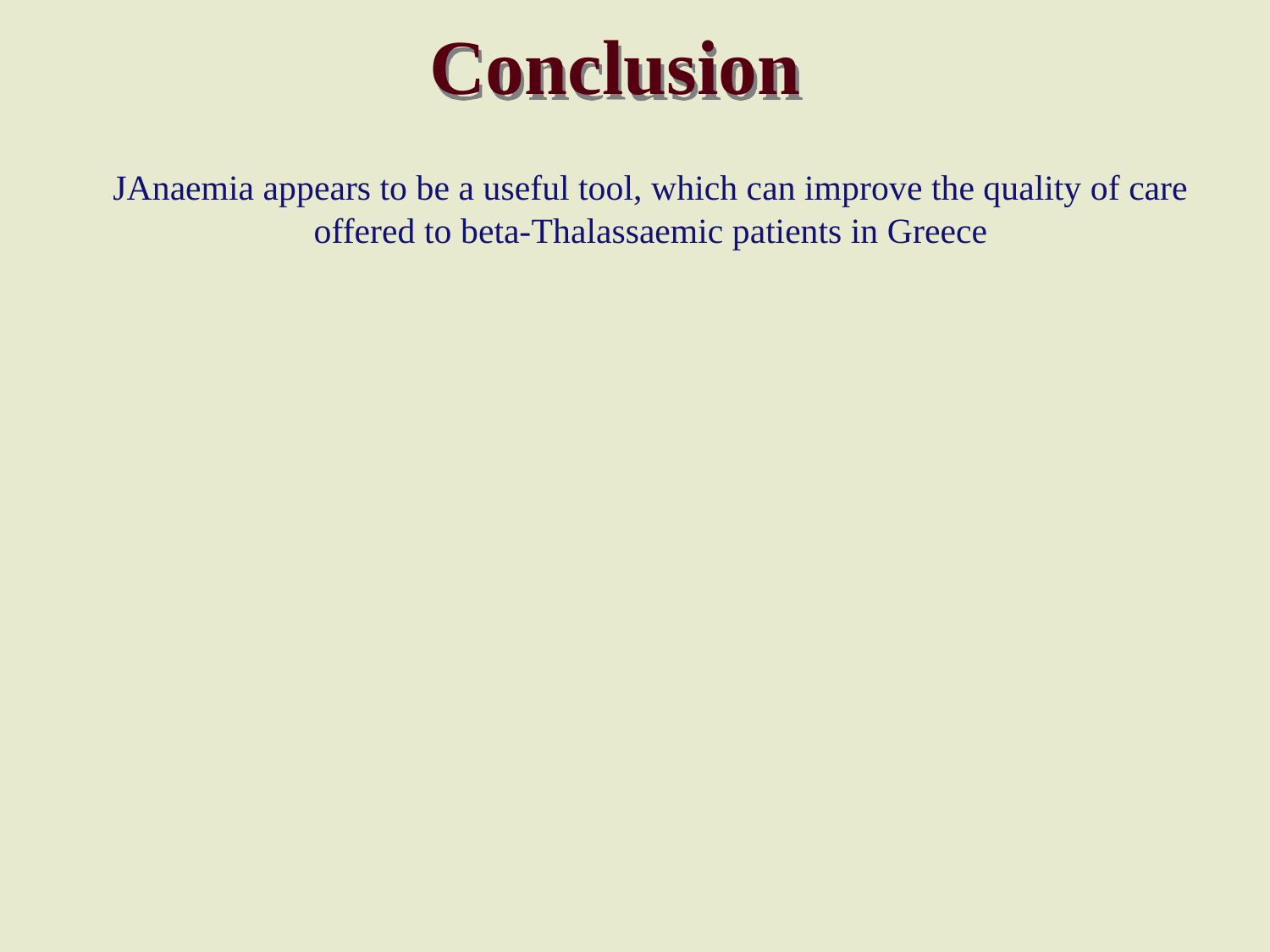

Conclusion
JAnaemia appears to be a useful tool, which can improve the quality of care offered to beta-Thalassaemic patients in Greece
